# Safety Assessment of Vitamin D and Its Photo-Isomers in UV-Irradiated Baker’s Yeast

**DOI:** 10.3390/foods10123142

**Published:** 2021-12-18

**Authors:** Tobias Schümmer, Gabriele I. Stangl, Wim Wätjen

**Affiliations:** 1 Postgraduate Course for Toxicology and Environmental Toxicology, Institute for Legal Medicine, University of Leipzig, Johannisallee 28, 04103 Leipzig, Germany; tschuemmer@gmail.com; 2 Institute of Agricultural and Nutritional Sciences, Martin-Luther-Universität Halle-Wittenberg, Von-Danckelmann-Platz 2, 06120 Halle/Saale, Germany; gabriele.stangl@landw.uni-halle.de; 3 Institute of Agricultural and Nutritional Sciences, Martin-Luther-Universität Halle-Wittenberg, Weinbergweg 22, 06120 Halle/Saale, Germany

**Keywords:** tachysterol, lumisterol, photoproduct, UV-irradiated food, vitamin D supplementation

## Abstract

Vitamin D deficiency due to, e.g., nutritional and life style reasons is a health concern that is gaining increasing attention over the last two decades. Vitamin D_3_, the most common isoform of vitamin D, is only available in food derived from animal sources. However, mushrooms and yeast are rich in ergosterol. This compound can be converted into vitamin D_2_ by UV-light, and therefore act as a precursor for vitamin D. Vitamin D_2_ from UV-irradiated mushrooms has become an alternative source of vitamin D, especially for persons pursuing a vegan diet. UV-irradiated baker’s yeast (*Saccharomyces cerevisiae*) for the production of fortified yeast-leavened bread and baked goods was approved as a Novel Food Ingredient in the European Union, according to Regulation (EC) No. 258/97. The Scientific Opinion provided by the European Food Safety Authority Panel on Dietetic Products, Nutrition, and Allergies has assessed this Novel Food Ingredient as safe under the intended nutritional use. However, recent findings on the formation of side products during UV-irradiation, e.g., the photoproducts tachysterol and lumisterol which are compounds with no adequate risk assessment performed, have only been marginally considered for this EFSA opinion. Furthermore, proceedings in analytics can provide additional insights, which might open up new perspectives, also regarding the bioavailability and potential health benefits of vitamin D-fortified mushrooms and yeast. Therefore, this review is intended to give an overview on the current status of UV irradiation in mushrooms and yeast in general and provide a detailed assessment on the potential health effects of UV-irradiated baker’s yeast.

## 1. Introduction

### 1.1. General Background

Vitamin D has a special role among vitamins. Given enough exposure to sunlight, the human body can synthesize the required amount by itself, and beyond. Daily doses by this endogenous synthesis can reach up to 10,000 international units (IU), while only 400 to 1000 IU are recommended as daily intake [1]. Therefore, its declaration as a vitamin is misleading, as the major source in humans can be its synthesis from precursors in the skin. Only in cases of insufficient exposure to UV-light of sufficient wavelength, an uptake from nutritional sources is required to prevent rickets in children and osteomalacia in adults. Other proposed activities of vitamin D include involvement in autoimmune diseases and respiratory diseases [2]. Several studies have shown anti-cancer activities of vitamin D, but also interactions with other hormone systems involved in reproduction and Alert-Fight-Defence response have been proposed next to calcium homeostasis [3,4,5,6].

**Figure 1:**
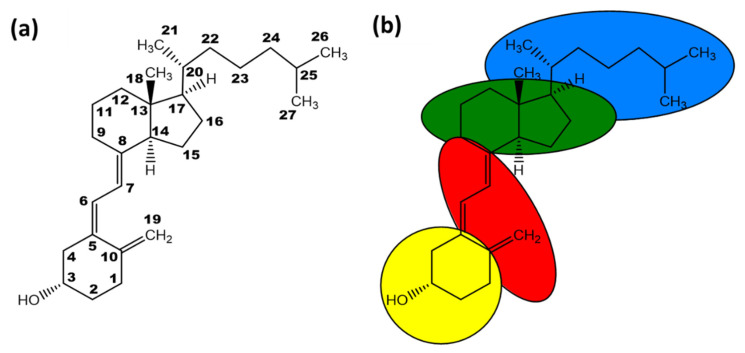
Structural elements of vitamin D_3_. (**a**) Structural formula of vitamin D_3_ with the canonical numbering of the carbon atoms. (**b**) The four structural elements of vitamin D_3_ are highlighted: A-ring (yellow), conjugated triene region (red), C- and D-ring (green), and sidechain (blue).

There are several vitamin D isoforms which all share the same core structure. The different isoforms show minor modifications in the side chain of vitamin D (see Figure 1).

**Figure 2:**
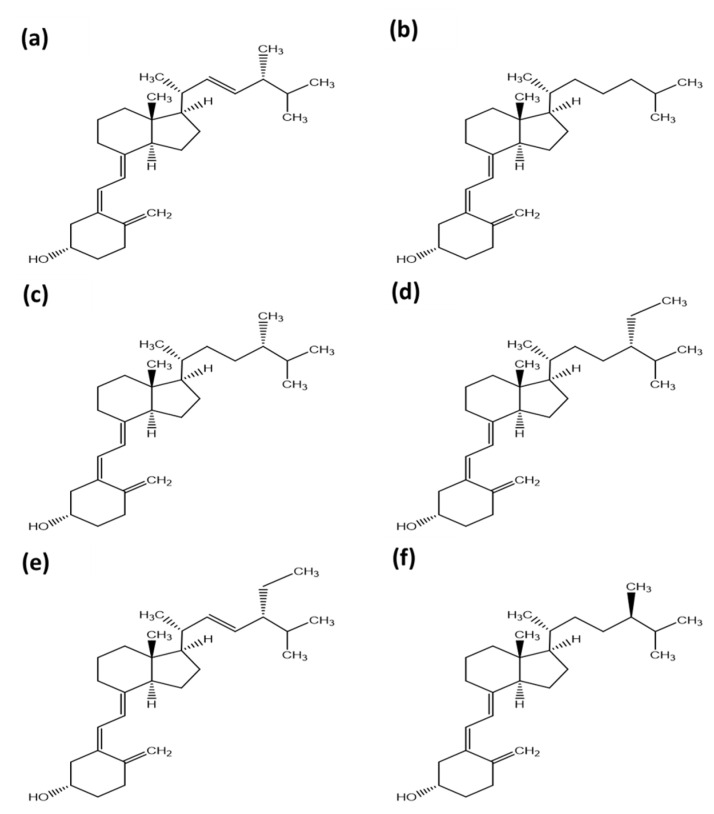
Isoforms of vitamin D: (**a**) Ergocalciferol or vitamin D_2_; (**b**) cholecalciferol or vitamin D_3_; (**c**) 22-dihydroergocalciferol or vitamin D_4_; (**d**) sitocalciferol or vitamin D_5_; (**e**) vitamin D_6_; (**f**) vitamin D_7_.

An overview of the chemical structure of currently known isoforms of vitamin D is given in Figure 2.

### 1.2. Vitamin D Synthesis and Activity

**Figure 3:**
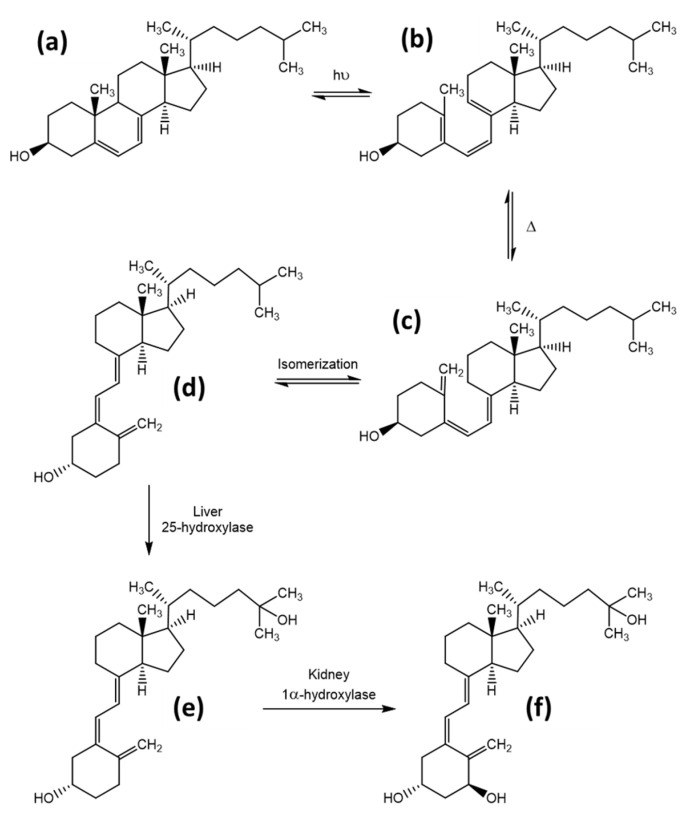
Synthesis pathway of the active hormonal form 1,25(OH)_2_D_3_ from 7-dehydrocholesterol. Breakage of the 9,10-bond within the B-ring of 7-dehydrocholesterol (**a**) by ultraviolet radiation forms previtamin D_3_ (**b**). The ring opening is followed by a rapid, thermally induced isomerisation by a [1,7]-hydrogen atom shift (**c**), followed by a spontaneous shift from the 6-*cis*-conformer to the 6-*trans*-conformer, resulting in vitamin D_3_ (**d**). These three reaction steps occur spontaneously without any enzyme catalysis, but at a low efficacy in the body. Hydroxylation at position C25 by 25-hydroxylase in the liver to 25(OH)D_3_ (**e**), and a second hydroxylation by 25(OH)D-1-hydroxylase in the kidney results in the active hormonal form, 1,25(OH)_2_D_3_ (**f**).

The role and synthesis pathway of vitamin D was discovered approximately 100 years ago. In 1919, Huldschinsky showed that UV-B light exposure (280–315 nm) can prevent and cure rickets in children. The same effect was also observed when certain fatty food sources, which had been exposed to artificial or natural UV-light, were applied to rachitic animal models. These food sources included linseed oil, whole milk, cod liver oil, and even liver and muscle tissue of vitamin D- and UV-light-devoid rats, where the tissues had been exposed to UV-light ex vivo [7]. However, the actual substance and its structure remained unknown until 1936 when Adolf Windaus established the molecular structure of calciferol and proposed the synthesis pathway of ergosterol/vitamin D_2_ in the body [7,8]. The synthesis of vitamin D involves both enzymatic as well as photosynthetic steps and will be explained in detail below for vitamin D_3_, although the same mechanism is utilized for all of the vitamin D variants. The precursor of vitamin D_3_ is 7-dehydrocholesterol, which can be obtained from nutritional sources or is produced enzymatically from the cholesterol precursors lathosterol or dehydrodesmosterol. UV radiation causes a ring-opening by cleavage of the 9,10-bond of the B-ring, forming previtamin D_3_ [9]. The specific ring opening occurs at the conjugated double bond in ring B. This double bond has a strong π to π* absorption between 250 and 310 nm, with a maximum absorption at 281 nm [10]. The optimal wavelength for an effective ring opening while giving the lowest rise to photo-isomer formation was observed at 295 nm [11]. The efficacy of this reaction is quite low, reaching only 2% to 3% after 20 to 30 min direct sunlight exposure (June, latitude of Boston) [12]. The ring opening is followed by a rapid, thermally induced isomerisation by a [1,7]-hydrogen atom shift, followed by a spontaneous shift from the 6-*cis*-conformer to the 6-*trans*-conformer, resulting in vitamin D_3_, also named cholecalciferol. After this step, the molecule is bound to the vitamin D-binding protein (DBP) and redistributed to the liver after its release from the skin into the blood stream [13]. In the liver, a first hydroxylation step occurs, resulting in 25-hydroxy-cholecalciferol, 25(OH)D. This step is catalysed in the endoplasmatic reticulum by several different proteins, all of which possess 25-hydroxylase activity. The most prominent enzymes are the microsomal CYP2R1 and the mitochondrial CYP27A1 [14,15]. The 25(OH)D_3_ is again released into the blood stream, and by itself has little biological activity due to its low affinity to the vitamin D-receptor (VDR), which functions as a transcription factor and mediates most of the vitamin D effects [16]. A second hydroxylation at position 1 results in the active form, 1,25(OH)_2_D. This reaction takes place predominantly in kidney mitochondria in the proximal tubular epithelial cells and is catalysed by 25(OH)D_3_-1-hydroxylase (also known as 1α-hydroxylase or CYP27B1) [17]. Synthesis of the active hormonal form is tightly controlled, which is stimulated by parathyroid hormone and inhibited by calcium, phosphate, and the fibroblast-growth-factor 23 (FGF23) [14]. An overview on the synthesis pathway is provided in Figure 3, modified from Agoston et al. [18].

In contrast to the other cholesterol-based steroid hormones, the presence of the direct precursor of 1,25(OH)_2_D_3_ is the rate-limiting step during synthesis. For other cholesterol-based hormones, the precursors are available in a millimolar range, while the serum concentrations of 25(OH)D_3_ and its precursors are only in the nanomolar range. The liver is able to adjust its enzymatic activity to produce 25(OH)D_3_ proportionally to the available substrate, and the reaction does not seem to be saturable in vivo [19]. After its synthesis, 1,25(OH)_2_D_3_ is distributed by the bloodstream. Approximately 85% of the 1,25(OH)_2_D_3_ are bound to the vitamin D-binding protein (DBP), approximately 15% are bound to albumin, and only marginal amounts (0.4%) are free in serum [20]. Only this non-bound form of 1,25(OH)_2_D_3_ is taken up by the cells to interact with the intracellular vitamin D receptor. VDR is a nuclear receptor that is activated when bound to 1,25(OH)_2_D_3_ and acts on the transcription of several hundred different genes [15,18,21]. Next to the classical target sites in the intestine, bone, and kidney, radioactive labelling has shown its presence in the anterior and posterior pituitary, skin epidermis with hair sheaths and sebaceous glands, reproductive organs such as the placenta, pancreatic B-cells, and defined groups of neurons, and glial cells in the brain and spinal cord. Furthermore, targets for 1,25(OH)_2_D_3_ have been identified in heart myocytes, stomach G-cells, oesophageal epithelium, thymus reticular cells, certain cells in the thyroid, trachea, and lymphatic nodules, kidney podocytes and macula densa cells, bone marrow reticular cells, osteoprogenitor cells, etc. [5]. A variety of gene products are influenced by 1,25(OH)_2_D_3_, including polyamine biosynthesis, lymphokine biosynthesis, and synthesis of calcium binding proteins. By its gene-regulating activities, 1,25(OH)_2_D_3_ has a strong impact on cellular growth and differentiation, as well as mineral homeostasis and the immune system. A detailed overview on target genes can be found in a review by Minghetti and Norman [22].

The 1,25(OH)_2_D_3_ exerts its action by binding to the VDR. VDR is the only known nuclear receptor in humans that is able to bind 1,25(OH)_2_D_3_ with high (subnanomolar) affinity. VDR is expressed ubiquitously, and its expression has been found in cancer cell lines, as well. Therefore, a role in cancer therapy is also discussed and resulted in the synthesis of a large variety of vitamin D analogues [18]. VDR is an endocrine receptor and has a mechanism such as glucocorticoid- or oestrogen-receptors. The ligand binding domain of VDR is structurally conserved and is comprised of 11 to 15 α-helices. Upon binding of calcitriol or an analogue with sufficient affinity for the binding site, VDR undergoes a conformational change, thereby causing both the dissociation of co-repressors of the ligand-binding domain, as well as the binding of co-activators [21]. In particular, the heterodimerisation with a retinoid X receptor and consequent binding of the N-terminal domain of VDR to vitamin D response elements in the DNA, regulate the transcription of target genes [23]. Co-activators can also attract chromatin-modifying enzymes to enable the binding to the vitamin D response elements.

Despite this enormous number of target sites for VDR, the main regulatory role of 1,25(OH)_2_D_3_ is the regulation of calcium and phosphate blood stream concentrations by increasing calcium and phosphate uptake from the gut across the intestinal mucosa [19]. The uptake mechanism of calcium from the gut includes both an active transport and a passive diffusion mechanism. The active transport mechanism involves TRPV6 (formerly named CaT1), which mediates the entry of calcium across the brush border. It was shown that 90% of the activity of TRPV6 is induced by 1,25(OH)_2_D_3_, and that low calcium and phosphate levels will enhance the formation of 1,25(OH)_2_D_3_ [24]. Other regulators of calcium and phosphate homeostasis next to 1,25(OH)_2_D_3_ are parathyroid hormone and fibroblast growth factor 23 (FGF23). All of these factors tightly control each other to maintain a constant calcium and phosphate plasma level. Parathyroid hormone is an 83 amino acid peptide hormone synthesised in the parathyroid gland. Its expression and release into the blood stream is induced via the calcium-sensing receptor at low calcium levels in the plasma. Upon release into the blood stream, parathyroid hormone prompts bone degradation to release calcium, providing a faster increase of calcium plasma levels compared with 1,25(OH)_2_D_3_. Therefore, it is not surprising that one of the main target organs of vitamin D is the parathyroid gland. The cells of the parathyroid gland show a high expression of VDR, and thus the lack of vitamin D can cause hyperparathyroidism, which will result in a loss of bone density due to the release of calcium from the bones after excessive parathyroid hormone release. This increased synthesis of parathormone would normally be suppressed by the 1,25(OH)_2_D_3_-VDR complex [3,6]. On the other hand, the parathyroid hormone induces the synthesis of 1,25(OH)_2_D_3_ by activating the expression of 25(OH)D-1-hydroxylase. On the other hand, FGF23 inhibits both the transcription of 25(OH)D-1-hydroxylase and the release of parathyroid hormone in the case of an elevated calcium and phosphate plasma level, while its own release is induced by 1,25(OH)_2_D_3_. Finally, 1,25(OH)_2_D_3_ acts on its own synthesis by a negative feedback loop on the transcription of 25(OH)D-1-hydroxylase [25].

### 1.3. Vitamin D Transport, Metabolism, and Elimination

Vitamin D and its hormone metabolites are highly lipophilic. This requires specific transport mechanisms for the delivery of these compounds. The endogenously produced vitamin D in the skin is bound by the vitamin D-binding protein (DBP) and after its dissociation can be taken up by target cells and intracellularly reach its target sites, predominantly VDR. However, the presence of an intracellular vitamin D-binding protein (IDBP) also increases the uptake and storage facility of the cells. Furthermore, it has also been shown that IDBP can modulate 1,25(OH)_2_D synthesis by influencing the transport of 25(OH)D to its mitochondrial target site and the upregulation of the catabolic CYP24 gene (see below) [26]. On the other hand, the uptake of vitamin D from nutritional sources is still not completely elucidated. While diffusion does play a part in the uptake, the majority of vitamin D is taken up by the active membrane transport. There are several membrane transporters that were shown to influence the vitamin D uptake, particularly cholesterol transporters and transporters for long-chain fatty acids [27,28]. Dietary forms are also taken up and distributed within lipoprotein particles [29,30,31].

**Figure 4:**
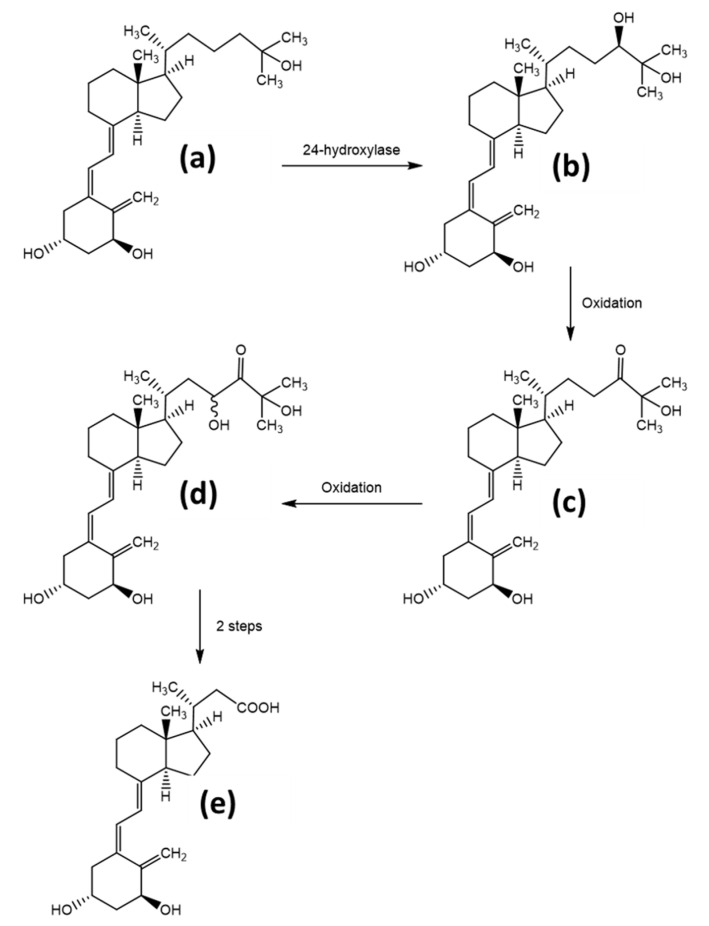
Main degradation pathway of 1,25(OH)_2_D_3_ in humans. The enzyme 24-hydroxylase (CYP24A1) activates 1,25(OH)_2_D_3_ (**a**) by initiating a hydroxyl-group at position 24, giving the metabolite 1,24R,25(OH)_3_D_3_ (**b**). The following oxidation of this hydroxyl group to the α-hydroxy ketone gives the catabolite 1,25(OH)_2_-24-oxo-D_3_ (**c**). Further oxidation of the sidechain at the C23 position results in 1,23,25(OH)_3_-24-oxo-D_3_ (**d**), followed by a two-step oxidative C-C bond cleavage resulting in the hormonally inactive catabolite calcitroic acid (**e**).

The metabolism and final elimination of the hormone form 1,25(OH)_2_D occur by a mechanism, which is common throughout all of the organisms. In a first step, the enzyme 24-hydroxylase (CYP24A1) activates the substrate by initiating a hydroxyl-group at position 24, producing the metabolite 1,24R,25(OH)_3_D. The following oxidation of this hydroxyl group to the α-hydroxy ketone gives the catabolite 1,25(OH)_2_-24-oxo-D. Further oxidation of the sidechain at the C23 position results in 1,23,25(OH)_3_-24-oxo-D. Finally, an oxidative C-C bond cleavage results in the hormonally inactive catabolite calcitroic acid. Calcitroic acid is eliminated through the bile and urine. The detailed mechanism, shown for vitamin D_3_, is given in Figure 4, modified from Agoston et al. [18]. Although the mechanism described above is the most utilized elimination pathway, alternative degradation pathways exist, which act both on the elimination of the active hormonal form, as well as the prevention of overproduction of the active form. Thereby, other products such as 24,25(OH)_2_D, 25,26(OH)_2_D, and 25(OH)D_3_-26,23-lactone are produced [32].

As with all of the hormones, the organism has a tightly controlled system to prevent any excess of the active metabolite. The first step of regulation is at the synthesis step. The activity of 1α-hydroxylase, which converts 25(OH)D_3_ to the active form 1,25(OH)_2_D_3_, is regulated both by calcium concentrations and a classical feedback loop of product concentration. Degradation of the precursor 25(OH)D is another mechanism utilized in the body, when the cytochrome P450-containing enzyme 24*R*-hydrolase (CYP24) converts 25(OH)D_3_ to 24*R*,24(OH)_2_D_3_, which is a less active metabolite, followed by further oxidation and elimination from the body [18]. In the case of an increased plasma concentration of 1,25(OH)_2_D_3_, the renal 25(OH)D-1-α-hydroxylase protein concentration is decreased and upregulation of 25(OH)D-24 hydroxylase (also termed 24-hydroxylase, 24*R*-hydroxylase or CYP24) is induced. This enzyme hydroxylates the side-chain of both 1,25(OH)_2_D and 25(OH)D [19]. Similar results of free 1,25(OH)_2_D acting as a negative feedback on vitamin D synthesis were also obtained in vitro for keratinocytes, albeit by a different mechanism as 24-hydroxylase, as the decrease of 1,25(OH)_2_D_3_ was observed even before the induction of 24-hydroxylase activity, pointing towards the regulation of 1α-hydroxylase [33].

### 1.4. Vitamin D Toxicity

In recent years, a raising interest in vitamin D both in scientific research as well as in general public has been observed (30). Various health benefits have been associated with a high intake of vitamin D, which gave rise to a strong increase in vitamin D toxicity, both acute and chronic. The currently recommended daily intake varies greatly, ranging from 400 IU in the UK up to 1000 IU by the International Osteoporosis Foundation. The tolerable upper intake level in both North America and Europe has been set to 50 µg or 2000 IU per day for children and 100 µg or 4000 IU for adults (31,32). However, the actual amount of vitamin D in the plasma is not measured. Instead, the serum concentration of 25(OH)D is taken as a substitute measurement. With the increasing general interest in vitamin D, substitution of vitamin D can result in vitamin D toxicity (VDT) due to (a) manufacturing errors, as well as (b) accidental and intended intake of high doses of vitamin D (1). Doses up to 100,000 IU daily have been recommended in public literature (33). Currently used diagnostic cut-off points for 25(OH)D to determine the vitamin D status of a patient are provided in Table 1.

The major hallmarks of VDT are hypercalciuria, hypercalcemia, elevated 25(OH)D concentrations above 150 µg/L, and usually normal or slightly increased 1,25(OH)_2_D concentrations [36]. The most prominent feature and easiest accessible biomarker of VDT is hypercalcemia. Symptoms of hypercalcemia are usually thirst and polyuria for mild symptoms. Hypercalciuria is strongly associated with hypercalcemia, as this is a strategy of the body to eliminate the unphysiological high serum concentrations of calcium. Other symptoms of VDT include muscle weakness, hypertension, neuropsychiatric disturbances, gastrointestinal upset, renal calculi, demineralization of the bone with simultaneous nephrocalcinosis, and, in extreme cases, renal failure, deposition of calcium phosphate crystals, cardiac arrhythmias by a reduced action potential, calcification of coronary vessels and heart valves, seizures and ultimately, coma and death [1,32,34,36].

It has been of major interest to find the causative agent of vitamin D toxicity. A detailed overview has been provided by Glenville Jones in 2008 [15]. In a wide variety of animal studies in different species, it has been found that the active hormonal form 1,25(OH)_2_D is hardly influenced by the presence of its precursors, which again demonstrates the high regulation of hormone synthesis in the liver and kidneys. Therefore, the precursors and metabolites of vitamin D have become of interest regarding their toxicity, including 25(OH)D, 24,25(OH)_2_D, 25,26(OH)_2_D, and 25(OH)D-26,23-lactone. With increasing doses of perorally applied vitamin D, the plasma concentrations of all these metabolites increase significantly, while the plasma concentration of 1,25(OH)_2_D_3_ is not influenced [4,32]. Due to the strong lipophilic character of all of these compounds, adipose tissue is seen as a storage compartment for vitamin D (even if the mobilization compared with the liver is rather low), as well as its precursors and degradation products. In the case of prolonged peroral supplementation, the adipose tissue can prevent early signs of toxicity. After saturation of the binding capacity of adipose tissue, however, toxicity can occur fast, and can retain even after peroral supplementation has been stopped due to the release of vitamin D from adipose tissues. As with other highly lipophilic compounds which can exert toxic effects, a rapid loss of adipose tissue can result in a rapid release of vitamin D, causing VDT [32]. Hypercalcemia as an indicator for vitamin D toxicity was seen for peroral doses of 650 ng/d in rats. Considering all of the animal studies, the safety margin at 375 nmol/L serum 25(OH)D could be confirmed. These results were also attested by anecdotal reports for both vitamin D_2_ and D_3_ toxicity [13,15].

The exact mechanism causing toxicity is still under investigation. This is especially interesting, as vitamin D toxicity is only found from peroral supplementation, but never from excessive exposure to sunlight. There are several conflicting theories regarding vitamin D toxicity, which were proposed by Jones in 2008 [15]:

1. Through an increased intake of vitamin D, the plasma 1,25(OH)_2_D concentrations, and consequently the cellular 1,25(OH)_2_D concentrations increase.
2. Vitamin D intake raises plasma 25(OH)D to concentrations that exceed the DBP binding capacity and “free 25(OH)D” enters the cell, where it has direct effects on gene expression by binding to VDR, although it has a lower binding affinity compared with 1,25(OH)_2_D.
3. Vitamin D intake raises the concentrations of many vitamin D metabolites, especially vitamin D itself and 25(OH)D. These compounds at high concentrations compete for the DBP binding capacity and cause the release of free 1,25(OH)_2_D, which enters the target cells and binds to VDR.

The mechanism proposed by Vieth in 1990 also proposes three features impacting VDT. These are the binding capacity of DBP, the level of residual 1α-hydroxylase, and the clearance capacity of the body for vitamin D and its metabolites. The binding capacity is of importance, as the free, not the bound form of 1,25(OH)_2_D is the active form. With high concentrations of 25(OH)D and other metabolites, the active form can get displaced from DBP, therefore exerting its hormonal activity after uptake into cells and binding to VDR [13]. Whether or not the IDBP can act as a buffer to prevent VDT has, to the knowledge of the author, not been assessed.

According to Marcinowska-Suchowierska et al. [36], abnormally high 25(OH)D and free 1,25(OH)_2_D are more likely compared with increased overall 1,25(OH)_2_D-concentrations due to the high regulation of 1,25(OH)_2_D synthesis. However, proof is still lacking for any of these theories to be discarded.

## 2. Other Isoforms of Vitamin D

The most important vitamin D isoform for human nutrition is vitamin D_3_ from animal sources. However, there are several other isoforms of vitamin D (D_2_, D_4_ to D_7_) which can be present in nutrition and can have relevant hormonal activity after their enzymatic activation. The order of activity of the vitamin D isoforms in humans is (excluding vitamins D_6_ and D_7_, as there are no available data on the activity of these vitamin D isomers compared with the other vitamin D isomers):

D_3_ ≥ D_2_ > D_4_ >> D_5_

The activity level of vitamin D_2_ compared with vitamin D_3_ is currently under discussion, e.g., in the US the prevalently supplemented vitamin D form is D_2_. While vitamin D_2_ and vitamin D_3_ for decades were considered to be of equal potency to increase serum calcium levels, recent findings suggest that the efficacy of vitamin D_2_ might be lower than vitamin D_3_. This is mostly assigned to different pharmacokinetics of the two isoforms. While doses of 50,000 IU vitamin D_2_ or vitamin D_3_ in human volunteers cause a similar increase of serum 25(OH)D in the first 3 days, the group receiving vitamin D_2_ showed a rapid decline back to the baseline within 14 days, whereas there was a prolonged increase and sustained 25(OH)D level in the vitamin D_3_ study group [37]. Similar results were also observed by a second study group [38], while most of the recent studies could not find a difference in activity [39,40]. A detailed assessment of the effect on the plasma concentrations of vitamin D_2_ and vitamin D_3_ after substitution for 8 weeks in healthy volunteers again showed that the effect of vitamin D_3_ is more pronounced, and that substitution with vitamin D_2_ can actually lead to an overall decrease of the total vitamin D plasma level [41]. A more detailed assessment of the biological activity of vitamin D_2_ is given in the following chapters.

Vitamin D_4_ has an activity of approximately 60% of the activity of vitamin D_3_. Its precursor 22,23-dihydroergosterol is found in all of the mushrooms, and the conversion to vitamin D_4_ is induced by exposure to UV-B light, as for vitamins D_2_ and D_3_. The amount of the precursor 22,23-dihydoergosterol has been tested in commercial samples of *Agaricus bisporus,* which had been irradiated by UV-B. The amount was found to be 42 to 95 µg/100 g dry weight [42]. This corresponds to approximately 10% of the ergosterol content in the same samples. Several studies have investigated the vitamin D_4_ content in mushrooms. Interestingly, there are reports stating that no vitamin D_4_ is detected in sunlight-exposed mushrooms, whereas there is a linear correlation between vitamin D_2_ and vitamin D_4_ in UV-B exposed mushrooms. The amount of vitamin D_4_ in the mushrooms can reach up to 18 µg/g dry weight, and the ratio of vitamin D_2_ compared with vitamin D_4_ after UV-light exposure is dependent on the wavelength and light intensity and is approximately 5:1 of vitamin D_2_ to vitamin D_4_ [43]. This is in contrast with Philips et al., who describes the detection of vitamin D_4_ in sunlight-exposed mushrooms also, and where the concentration of vitamin D_4_ could be similar or even greater than the concentration of vitamin D_2_. However, the results of Philips et al. have been questioned and attributed to insufficient separation or coelution of different vitamin D isomers [44]. The average amount of vitamin D_4_ found in mushrooms was 5.2 µg/100 g fresh weight, and vitamin D_4_ could be detected in 18 out of 38 samples of commercially available mushrooms. The analytics of vitamin D_4_ are not straightforward, as high pressure liquid chromatography (HPLC) methods are often not able to univocally identify the isomer, since the corresponding standards are not yet commercially available and discriminate between the different isoforms [42,44]. An additional aspect that has to be kept in mind for all of these measurements is the lack of analytical standards for many of the vitamin D photo-isomers, making the identification and quantification of these compounds even more complex.

To gain a better understanding of vitamin D_4_ activity and kinetics in rats and chicks, a tritium-labelled vitamin D_4_ was synthesised starting from ergosterol, as reported by Luca et al. in 1968. The activity of vitamin D_4_ was found to be approximately 60% of D_3_ in weanling male rats and about 20% in chicks. For the determination of the vitamin D_4_ distribution, rats were maintained on a vitamin D-deficient diet for 3 to 4 weeks until they started to bare clinical signs of vitamin D deficiency, mostly low serum calcium levels and growth retardation. Tritium-labelled vitamin D_3_ or D_4_ was administered to the animals by single gavage dosing. The animals were sacrificed 4 to 48 h after application and the distribution of the radiolabelled vitamins was determined by combustion analysis. The most prominent difference in pharmacokinetic behaviour between vitamin D_3_ and vitamin D_4_ in rats is a faster elimination of vitamin D_4_ from bone, blood, muscle, and kidney compared with vitamin D_3_. The highest concentrations of vitamin D_4_ in rats were observed in the intestinal mucosa and bone cells [45].

A similar approach was also used for the assessment of vitamin D_5_ by Napoli et al. in 1979. In this study, radioactive variants of vitamin D_2_, D_3_, and D_5_ were compared in vitamin D-deficient rats. After complete synthesis, tritium-labelled vitamin D isomers were utilized to determine the effects on intestinal calcium transport, bone calcium mobilization, and mineralisation of rachitic cartilage. For vitamin D_2_ and D_3_, serum calcium elevations were observed for doses of 12.5 ng/d and above, while no effect was observed for 2.5 ng/d compared with the control group receiving 1,2-propanediol. On the other hand, 2.5 ng/d were already sufficient in increasing the intestinal transport of calcium significantly. For vitamin D_5_, doses of 2500 to 5000 ng/d yielded similar results to those of 25 ng/d vitamin D_3_. Saturation of calcium uptake from the intestine was already observed for 1000 ng/d vitamin D_5_. These results showed about 80-fold lower activity in intestinal calcium transport, and about 100- to 200-fold lower activity in bone. A similar extent (180-fold) was also observed for the induction of vitamin D toxicity of D_5_ compared with D_3_. The authors concluded that the single-point substitution of an ethyl group at position 24 was responsible for this huge effect. A single substitution of an ethyl group at the same position has a lower effect, as seen for vitamin D_4_, while the introduction of a 22,23-ene seems to partially offset the detrimental effect of the methyl group at position 24 [46].

## 3. Products and Site Products in Mushrooms and Yeast after UV Irradiation to Induce Vitamin D_2_ Formation

### 3.1. Baker’s Yeast (Saccharomyces cerevisiae)

Baker’s yeast (*Saccharomyces cerevisiae*) belongs to the group of fungi, its cell membranes contain a high amount of ergosterol. Ergosterol regulates plasma membrane fluidity (such as cholesterol in animal cells), biogenesis, and function. Ergosterol synthesis is one target to treat fungal infections in humans due to the absence of ergosterol synthesis [47]. Ergosterol in *S. cerevisiae* is present as bound to the membrane or intracellularly, where it is involved in the regulation of ergosterol synthesis. Ergosterol acts as the precursor for vitamin D_2_ synthesis, similar to 7-dehydrocholesterol in animal cells for vitamin D_3_ synthesis. Depending on the strain and growth conditions, ergosterol can make up to almost 35 mg/g dry cell weight in *S. cerevisiae* [48]. These high amounts of ergosterol next to their ubiquitous use in food products make *S. cerevisiae* an ideal target for UV irradiation to increase the vitamin D_2_ content in food. This conversion has been studied in mushrooms over several decades. Therefore, a large quantity of literature exists for mushrooms, while the available literature on the irradiation of *S. cerevisiae* is not as comprehensive yet. To obtain a detailed picture on the potential effects of UV irradiation for vitamin D_2_ production in yeast, the literature on mushrooms will be included extensively in this review.

### 3.2. Yield of Vitamin D_2_ after UV Irradiation of Mushrooms and Yeast

Edible mushrooms can be a source of vitamin D_2_, as they contain ergosterol, which can undergo the same ring-opening mechanism and isomerisation reactions as 7-dehydrocholesterol, described in Chapter 1. First studies on the vitamin D_2_ content of mushrooms were already performed in the 1930s, but systematic assessments were only started almost 60 years later [49]. Ergosterol is a major compound of the fungal plasma membrane, and it was found that UV-B irradiation increases the vitamin D_2_ content in mushrooms and yeast [50,51,52]. Commercial utilisation of the method was initiated soon after that discovery, including attempts to gain an intellectual property for specific irradiation methods [53,54,55]. The ergosterol content in wild mushrooms is between 0.6% and 0.7% of the dry weight, which is higher than in yeast, thus providing enough substrate for conversion to vitamin D_2_. It was also shown that the vitamin D_2_ content of wild grown mushrooms can be very high, although there is a large variation in content both regarding species, parts of the mushroom, and also between individual fruiting bodies [56]. Cultivated mushrooms are normally devoid of vitamin D_2_, as they are not exposed to sunlight but are rather grown indoors [57]. This deficiency can be overcome by the aforementioned UV irradiation. However, the efficacy of the conversion by UV-B light is low in whole fruiting bodies due to the limited penetration depth of the UV-B light and an unfavourable surface-to-volume ratio of the budding fruits, reaching a molar conversion rate of 2% to 3%, similar to conversion rates seen in human skin [58]. Nevertheless, this is sufficient to cause an increase of vitamin D_2_ by more than 10-fold in the dried mushrooms. The efficacy of the irradiation can be improved even further if the mushrooms are dried, grinded, and exposed to UV-light when suspended in ethanol. The increase in shiitake mushrooms is more than 11,000%, while it is more than 4500% in oyster mushrooms when applying this method. It was further speculated that this procedure may further prevent oxidation and photodegradation, increasing both yields and preventing the production of side products. Unfortunately, the potential production of side products was not evaluated in this study [59]. The exact yield depends on a wide variety of parameters, including wavelength, time of exposure, and light intensity. An overview of the published results for different mushroom species and reaction conditions is provided in Table 2. In this table, only the highest yield for each mushroom species and chosen irradiation condition in each publication is provided together with the respective irradiation conditions. The summarized results show that there are tremendous differences regarding yield, based on the type of mushroom, state of mushroom at irradiation (fresh, sliced, dried resuspended in ethanol), as well as irradiation wavelength and intensity. Some results need to be treated with caution, as the irradiation dose reported by Teichmann et al. [60] is two orders of magnitude larger than most of the other used irradiation doses. Furthermore, the yields obtained by Guan et al. [57] are extremely high, especially given the short exposure time. The accuracy of the detection method has to be questioned in this case.

Vitamin D_2_ content was determined using HPLC in all cases. Additional factors influencing ergosterol conversion to vitamin D_2_ are temperature, moisture content, and most prominently the spectral distribution of the utilized UV lamps. While the increase of vitamin D_2_ can be highly significant, the conversion is still so low that there is little effect on the overall mushroom ergosterol content, mostly being around 2% to 3%. A linear relationship of the UV-B dose and vitamin D_2_ concentration was determined by Kristensen et al. for doses up to 1000 mJ/cm^2^, after which a non-linear behaviour with decreased conversion is observed in white button mushrooms (*A. bisporus*) during the growth phase [62]. This experiment shows that vitamin D_2_ enrichment can already be done during the growth phase, thus limiting the post-harvest processing steps while still exerting the potential health benefits. Furthermore, it is recommended to irradiate the mushrooms daily to increase the vitamin D_2_ content. Due to the fast growth rate of mushrooms, the amount of compounds formed during single-day irradiation diminishes very fast during growth, and using tube UV-B irradiation was shown to be more efficient than natural sunlight [62].

To determine the optimal irradiation conditions, a response surface methodology was applied for the conversion in powdered white button mushrooms. Three independent variables were taken, exposure time, ambient temperature, and irradiation sensitivity to determine the reaction conditions for the highest vitamin D_2_ yield [66,70]. A similar approach was utilized by Jasinge and Perera when performing a two-way ANOVA analysis utilising temperature and moisture content as main effects on vitamin D_2_ yield by irradiation, with these two parameters showing a positive linear correlation. A linear increase of vitamin D_2_ was determined under all of the reaction conditions for irradiation times of up to 40 min in four different types of mushrooms [71]. Interestingly, the wavelength or wavelength spectrum of the light sources were not considered to have a significant impact. Moreover, not only the yield of vitamin D itself is the desirable factor when applying UV-light to mushrooms. Furthermore, the negative effects on mushroom quality always have to be considered when applying UV-light, as discoloration, texture changes, and moisture loss due to evaporation will negatively impact the quality, turning the mushrooms undesirable for customers [66,70]. This also limits the options for producers who want to sell fresh or minimally unprocessed mushrooms, as for many studies, mushrooms had been partially or completely dried, grounded, sliced or lyophilised [63]. Therefore, a direct comparison of the results in Table 2 is not feasible for all of the determined yields.

Interestingly, for some mushroom types, cold storage seems to have a similar or even preferential effect on vitamin D_2_ formation. While initially only intended to determine the stability of vitamin D_2_ after UV-C irradiation, Guan et al. noticed that the vitamin D_2_ content in the stem of brown button mushrooms (*A. bisporus*) remained increasing after irradiation at cold storage over the course of 14 days, while the vitamin D_2_ content in the caps of both brown and white button mushrooms decreased initially, but remained stable after 7 days [57]. Overall, it is evident that the yield of UV irradiation of mushrooms needs to be optimized for each type, and thus a direct transfer of setup for irradiation in yeast is not possible.

### 3.3. Tachysterol and Lumisterol as Major Photoproducts Generated by UV Irradiation during Vitamin D Synthesis

**Figure 5:**
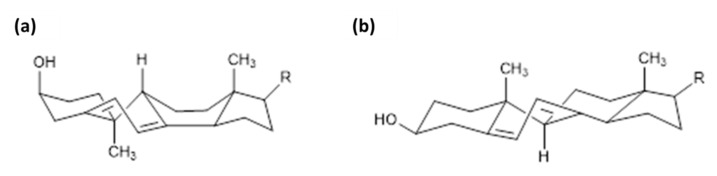
Configurations of lumisterol (**a**) and provitamin D (**b**).

Next to the described canonical reaction to form vitamin D, there are several other known products caused by photoinduced ring opening after UV-light exposure and photoinduced isomerisation reactions of intermediate and end-products during vitamin D generation. The first of these products is the 9,10 antiisomer of ergosterol, which is termed lumisterol. Lumisterol has a 9α,10β configuration, in contrast to the 9β,10α configuration of ergosterol. An important conformational difference between these two isoforms is the “boat”-form of the C-ring. A comparison of these two structures is shown in Figure 5. Lumisterol is formed by ring-closure after the UV-light-induced ring opening. The distribution of isomers after ring-closure is strongly temperature dependent, showing almost exclusively lumisterol (the 9-10 antiisomeric compounds) at 0 °C. At temperatures between 20 and 80 °C, the ring-opening reaction is strongly favoured due to the fast [1,2,3,4,5,6,7] H-shift of provitamin D to result in vitamin D [10].

**Figure 6:**
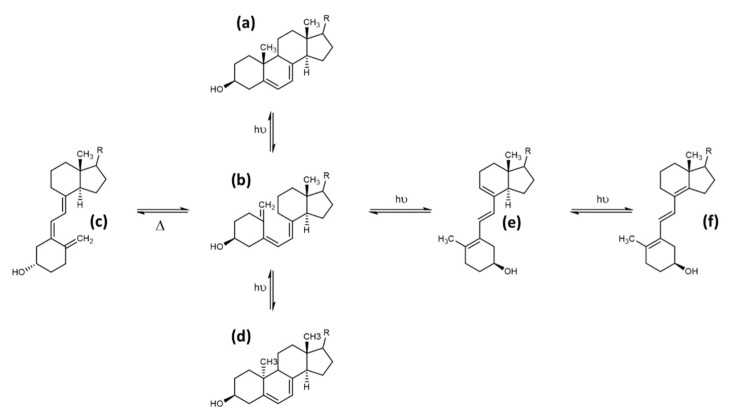
UV B-induced transformations of provitamin D to previtamin D, and consequently to vitamin D, lumisterol, and tachysterol. Provitamin D (**a**) is transformed by UV-light to previtamin D (**b**), followed by a thermally activated trans isomerisation to generate vitamin D (**c**). Alternative phototoinduced pathways are the formation of lumisterol (**d**) from previtamin D by ring-closure or the formation of tachysterol (**e**), and, after a second photoinduced isomerisation, iso-tachysterol (**f**).

The second common side product of the photolytic ring opening is tachysterol, which is the result of the Z ↔ E photoisomerisation reaction, resulting in the 6-7 E isomer. This photoisomerisation can also produce iso-tachysterol and occurs preferably at room or body temperature [72]. Tachysterol has structural similarity to vitamin D_2_, with its A-ring rotated by 180°, and a shift of the triene region in order for the double bonds to be located both within the A- and C-ring. The position of the double bond in the C-ring distinguishes tachysterol (C8-C9) from iso-tachysterol (C8-C14) [69,72]. The structures of provitamin D, previtamin D, lumisterol, and tachysterol were determined in the 1950s. In addition, the interconversion pathways defined in the 1970s also provide the exact quantum yields of the individual photochemical reactions [73,74] (Figure 6).

**Figure 7:**
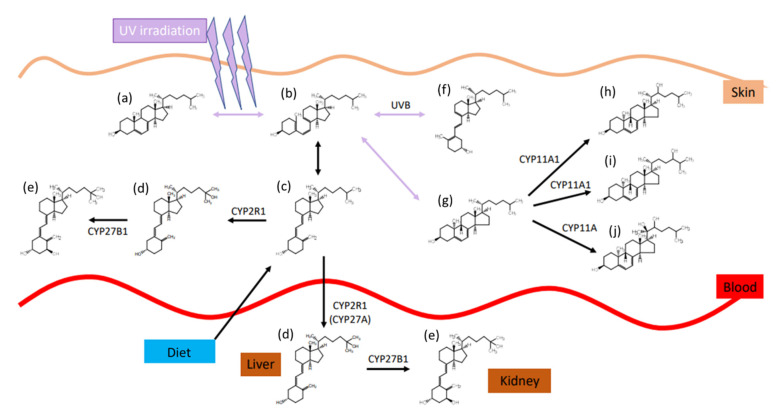
UV B-induced transformations of provitamin D to previtamin D, and consequently to vitamin D, lumisterol, and tachysterol. Provitamin D (**a**) is transformed by UV-light to previtamin D (**b**), followed by a thermally activated trans isomerisation to generate vitamin D (**c**). Enzyme-catalysed reactions in either skin or liver and kidney result in the formation of 25-hydroxyvitamin D (**d**), and finally 1,25-dihydroxyvitamin D (**e**). Alternative phototoinduced pathways are the formation of tachysterol (**f**) from previtamin D or the formation of lumisterol (**g**) by ring-closure. Further enzyme-catalysis of lumisterol results in the formation of 22(OH)lumisterol (**h**), 24(OH)lumisterol (**i**), and 20,22(OH)_2_lumisterol (**j**).

Tachysterol possesses no relevant chemical reactivity. It has a large extinction coefficient, and therefore its physiological function was estimated to prevent excessive UV- light-induced previtamin D formation. Tachysterol shows stronger photoproduct formation compared with vitamin D, and therefore could be a major alternative degradation pathway for vitamin D. Tachysterol can also act as a precursor for previtamin D when it is irradiated at wavelengths between 315 to 340 nm. This would allow the production of vitamin D even in winter or in the morning or evening at higher latitudes during summertime [75]. In a study using human skin samples, Holick et al. determined the formation of previtamin D_3_, tachysterol, and lumisterol upon UV-light exposure (Figure 7). After 15 min of exposure of the skin, previtamin D_3_ was the main photoproduct, and reached its maximum 15% of the initial 7-dehydrocholesterol concentration in the skin prior to the irradiation after 30 min. Longer exposure times resulted in the formation of tachysterol, which reached its maximum concentration of 5% of the initial 7-dehydrocholesterol concentration after 1 h. Lumisterol concentrations could reach up to 50% of the initial 7-dehydrocholesterol concentration in the skin after steady irradiation for 8 h (results from Type III skin). Irradiation times increased significantly with the higher pigmentation, but the maximum amount of 15% previtamin D was always reached regardless of skin pigmentation. The same effect was observed when adjusting light intensity from equatorial exposure to radiation common for higher latitudes. Therefore, the formation of tachysterol and lumisterol was proposed as a mechanism to prevent vitamin D toxicity from excessive previtamin D formation. The binding affinities of the vitamin D-binding protein for tachysterol and lumisterol are low compared with the binding affinity for vitamin D. This points towards a low mobility of these two compounds, preventing increased systemic concentrations and exposure of these side products [76].

However, it was shown in an animal experiment, that high doses of lumisterol (2 mg/kg b.w., 4 weeks) can enter the body, induce the formation of vitamin D_2_, reduce the levels of 25(OH)D_3_ and calcitriol, and strongly impact the expression of genes involved in the degradation and synthesis of bioactive vitamin D [77].

Similar results on the formation of lumisterol and tachysterol were also observed in irradiated whole fruiting bodies of *Pleurotus ostreatus* when exposed from the lamella side. After 1 h of exposure, lumisterol derived from ergosterol reached levels of approximately 50% of the generated ergocalciferol, while the level of tachysterol was at about 25%. Similar effects were also observed for the photoproducts of 22-dihydroergocalciferol, but these side products were present at a much lower level [58]. A detailed assessment on the formation of side products derived from both ergosterol and 22,23-dihydroergosterol has been published by Wittig et al. [44]. In this study, 10 different vitamin D_2_- and D_4_-related compounds (and vitamin D_3_ as internal standard) were determined by HPLC to assess the production upon irradiation of oyster mushrooms. Under the given reaction conditions of this study, the sum of all the vitamin D_4_ compounds reached approximately 20% of the sum of all the vitamin D_2_ compounds. The yields of the different photoproducts from the respective precursors were approximately 55% vitamin D, 21% lumisterol, and 12% of previtamin D and tachysterol, respectively. Tachysterol and lumisterol derived from vitamin D_2_ reached amounts of 30 and 50 µg/g dry weight of mushrooms. However, these yields were achieved under harsh treatment conditions during sample preparation prior to the analytic HPLC, making the bioavailability of these amounts from nutrition unlikely.

The ratio between different photoreaction products is strongly dependent on the irradiation wavelength. While shorter wavelengths (248 to 254 nm) favour the generation of tachysterol, the major products at wavelengths >305 nm are lumisterol and provitamin D [78]. The optimal wavelength for conversion of provitamin D to previtamin D is 295 nm, which is also the minimum for the formation of tachysterol and lumisterol [11]. Therefore, adjustment of both the wavelengths as well as the duration of UV-light exposure are utilized to prevent the formation of lumisterol and tachysterol [69]. Furthermore, it was found by Chen et al. that the side chain length and composition of the precursors influence the actual distribution of the side products during UV irradiation. Longer chains and the presence of a tertiary alcohol moiety on the side chain at C20 do facilitate a faster conversion from the previtamin D to vitamin D [72].

### 3.4. Other Photoproducts Generated by UV Irradiation during Vitamin D Synthesis

Additional side products of UV irradiation next to lumisterol and tachysterol, termed “overirradiation products” were observed in mushrooms at the beginning of the 1970s [11]. The structural determination of these compounds as well as a first assessment of their toxicity was performed by several study groups, mostly based in the Netherlands [10,79,80].

**Figure 8:**
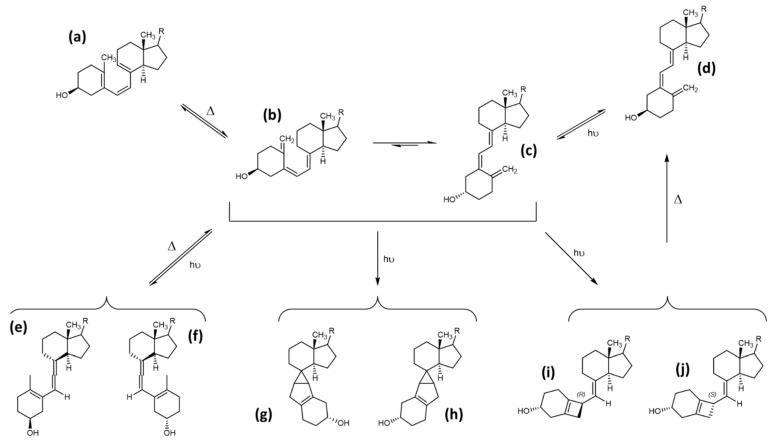
Isomerisation reactions and photoproducts of vitamin D, based on [10]. After UV irradiation, the cleavage product (**a**) rapidly isomerizes to previtamin D (**b**), which after a second isomerisation step forms vitamin D (**c**). The thermally induced reaction step from previtamin D to vitamin D involves a short-lived intermediate state of a 5,6-cis vitamin D, which can form several photoproducts. Depending on the photochemistry of the excited triene region, the photoproducts are the isomers of a 1,2,4-triene photoproduct ((**e**) and (**f**)), the suprasterols I and II ((**g**) and (**h**)) or the two isomers of a cyclobutene photoproduct ((**i**) and (**j**)). Both vitamin D and the cyclobutene photoproducts can form 5,6 trans-vitamin D (**d**).

The thermally induced reaction step from previtamin D to vitamin D involves a short-lived intermediate state of a 5,6-cis vitamin D, which rapidly isomerizes to vitamin D. Under UV irradiation, the *cis*-state can form several photoproducts due to the excitable state and conformational properties of the conjugated triene region. The maximum absorption of the conjugated triene region is at 260 nm. There are three possible planar conformations of the hexa-1,3,5-triene system, which can, upon excitation, form either a cyclobutene, a 1,2,4-triene or a [3,1,0] -bicyclohexane photoproduct. The first identified photoproducts, named suprasterols I and II (g and h in Figure 8), were bicyclohexane [3,1,0] derivatives. Due to steric hindrance, the ring-opening and reformation of the conjugated triene region is not possible for the suprasterols, making their formation irreversible. Another directly irreversible photochemical reaction is the formation of cyclobutene photoproduct (i and j in Figure 8). Upon thermal activation, this structure can open again, forming 5,6 *trans*-vitamin D (Figure 8). The 5,6 *trans*-vitamin D can also be formed in the presence of iodine and photoactivation, while the reverse reaction back to vitamin D only requires photoactivation. The last possible variant is the 1,2,4-triene photoproduct (Figure 8). This reaction can be reversed by thermal activation, likely due to the high energetic state of the electronic configuration of the neighbouring double bonds directly associated to a cyclohexane [10]. All of these products have been identified and spectroscopically characterized, but no exact statements can be made on their occurrence in nutrition upon natural or artificial UV irradiation of mushrooms or yeast.

**Figure 9:**
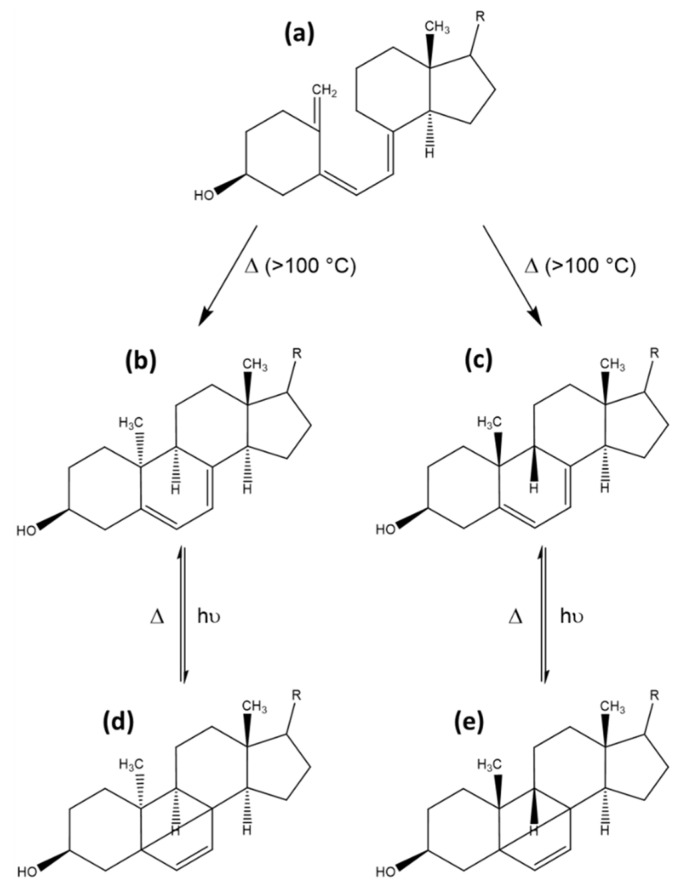
Synthesis pathway of pyrocalciferol and isopyrocalciferol and their respective photoproducts. Previtamin D (**a**) can undergo an irreversible ring-closure reaction at temperatures higher than 100 °C resulting in either a 9α, 10α confirmation (pyrocalciferol (**b**)) or a 9β, 10β confirmation (isopyrocalciferol (**c**)). Further UV irradiation results in the formation of the bicyclo [2,2,0] exene derivatives photopyrocalciferol (**d**) and photoisopyrocalciferol (**e**) in a reaction that can be reversed thermically.

Other side products can also be observed, produced at higher temperatures of 100 to 180 °C, irrespective of photoactivation. These products are formed by triene ring closure reactions of previtamin D, yielding 9–10 syn-isomers aptly named pyro- and isopyrocalciferol. The conformations of these calciferols are either 9α, 10α for pyrocalciferol or 9β, 10β for isopyrocalciferol. The chemical structures are rather contorted compared with provitamin D or lumisterol, and upon irradiation these compounds can form their corresponding bicyclo [2,2,0] hexene derivatives This reaction is rare due to a low quantum yield. The formation of these photocyclization products is thermically reversible. However, ring-opening of the B-ring back to the previtamin D is not possible due to steric hindrance [10]. An overview of the reaction scheme of pyrocalciferol and isopyrocalciferol is provided in Figure 9. Given the required reaction conditions, the formation of these compounds seems unlikely under physiological conditions. However, frying and cooking of food containing previtamin D can cause significant formation of pyro- and isopyrocalciferol [44]. A loss of approximately 10% of vitamin D during household cooking has been observed, although it is not clear if the loss can be attributed completely to the formation of pyro- and isopyrocalciferol [81].

One additional group of side-products formed under constant irradiation that cannot be reversed back to the precursor substances are 7,7′*bis*-cholestadienols formed from 7-dehydrocholesterol under strong UV irradiation, albeit with a small quantum yield. This formation has been observed at room temperature and below, and the resulting photoproducts are highly insoluble and are present in at least three distinct isoforms, depending on the location of the remaining two double bonds after the formation of the C7-C7′ carbon bond. However, the formation of these products has only been observed in ethanol at 0 °C and at higher concentrations of the precursor molecule [10]. Therefore, it is questionable if this product is produced at significant amounts under physiological conditions.

The formation of side products may fulfil an important biological function. As stated before, vitamin D toxicity cannot be caused by an excessive exposure to sunlight. This may be due to a self-regulation mechanism based on the intrinsic photochemical and photophysical properties of the vitamin D photo-isomers, which results in the quenching of previtamin D production under prolonged UV-light exposure. This regulation adapts within seconds and is not influenced by pigment formation [75].

UV irradiation can also cause an effect on the composition of other nutritional components. While not the focus of this study, it should be mentioned that the irradiation was shown to influence the linoleic acid content. This is likely caused by peroxidation of the fatty acid after release of hydroperoxides upon destruction of the mycelium [58]. The effects on the antioxidant capacity and polyphenol levels in UV-light-treated mushrooms were also tested in one case [64]. While a general effect on these compounds could be seen in this study, there were no tendencies observable according to the publication. In addition, it has been proposed in the past that UV-light exposure does not cause a compositional change or toxicological effect in mushrooms beyond an increase of the vitamin D_2_ and vitamin D_4_ content [42,65,82]. It was specifically shown that there is no effect on the content of the vitamins C, B_6_, riboflavin, niacin, and pantothenic acid [65].

## 4. Bioavailability of Vitamin D_2_ from Fungal Nutritional Sources

To gain an understanding of the potential effects of side products or contaminants of UV irradiation to produce vitamin D in yeast and mushrooms, it is inevitable to understand the bioavailability and efficacy of vitamin D_2_ provided by these enriched nutritional products. Naturally, the uptake and metabolism of vitamin D_2_ can only act as a proxy for the uptake of other substances present in mushrooms and yeast, as there are currently no studies available (to the knowledge of the author) that determine the uptake, distribution, and metabolism of vitamin D_2_ and all its UV-light-induced precursors, metabolites, and side products in an animal model.

The effect of vitamin D_2_ supplementation using UV-irradiated fungi has been tested in vivo by several groups. An overview on this topic was provided by Cardwell et al. [83]. The very first to show the uptake of vitamin D_2_ from mushrooms in humans were Outila et al. in 1999 [84]. In this study, 27 volunteers received either 14 µg/d ergocalciferol from winter mushroom (*Craterellus tubaeformis*), ergocalciferol supplementation of 14 µg/d or no kind of supplementation. All of the included participants had serum levels of 25(OH)D below 60 nmol/L before inclusion into the study. After 3 weeks, there was an approximate 1.5-fold increase in serum 25(OH)D concentration in the study groups receiving either mushrooms or supplementation, whereas there was no change for the group which did not receive any kind of supplementation. The mushrooms were lyophilised and homogenised before application, which likely was responsible for the high bioavailability of vitamin D from the biological matrix. A similar study was performed and reported by Urbain et al. in 2011, using a very high amount of vitamin D. In this study, 26 participants were assigned to one of three different study groups. Next to the control group, participants either received UV-B treated mushrooms or a vitamin D_2_ supplementation for 3 weeks by a weekly portion of mushroom soup, containing 700 µg vitamin D_2_. All of the three study groups were monitored for the study periods, as well as 2 additional weeks as follow-up. The results showed that as early as 2 weeks after initiation of the study, a significant increase of serum 25(OH)D was observed compared with the placebo group. For each participant, a significant effect was even observed after 1 week. For the short study period, an increase of 0.5 nmol/L 25(OH)D for every 2.5 µg of vitamin D was observed. Furthermore, a positive effect on secondary hyperparathyroidism was observed in the participants presenting clinical signs [67].

Contrasting results were obtained in a study performed by Mehrotra et al., where 43 prediabetic, vitamin D-deficient adults with a body mass index >25 received either daily doses of 100 g cooked mushrooms containing 15 or 100 µg vitamin D_2_ or peroral supplementation of vitamin D_2_ of 15 or 100 µg. After 16 weeks, only low effects on serum 25(OH)D were observed in the group receiving the fortified dishes, while there was a significant impact in the groups receiving supplementation. The low effect of the fortified dishes could be assigned to the loss of vitamin D_2_ due to the cooking process, as well as a lower bioavailability of vitamin D_2_ when in a biological matrix. The health status of the participants might also be a contributing factor [85]. Similar effects have also been determined in a study comparing the application of either 15 µg vitamin D_2_ from mushroom powder or 15 µg of vitamin D_3_, always in capsule form, and compared against the placebo. While an increase of the serum 25(OH)D_2_ level was observed for the group receiving vitamin D_2_, the overall impact on serum 25(OH)D was not affected by this supplementation. On the other hand, vitamin D_3_ was able to significantly increase the overall status by 55% [86]. One can even assume a trade-off between 25(OH)D_2_ and 25(OH)D_3_ in serum. In a human study involving 38 healthy volunteers, vitamin D_2_ was provided in varying concentrations from UV-light-treated mushrooms or a supplement. While there was a significant increase in 25(OH)D_2_ in serum, the overall 25(OH)D level was not affected, as the 25(OH)D_3_ level decreased accordingly [87]. While one can speculate on a stronger degradation and elimination effect of 25(OH)D_2_ on 25(OH)D_3_, another likely explanation is a lowered release of vitamin D_3_ or 25(OH)D_3_ from adipose tissues, which can act as storage in times of high vitamin D availability, e.g., in the summertime or at times of high nutritional supply.

Jasinghe et al. were the first to report on the bioavailability of vitamin D_2_ from UV-irradiated mushrooms in an animal model [88]. In this study, male vitamin D-deficient Wistar rats were fed a diet containing lyophilized, powdered Shiitake mushrooms for 4 weeks. The first group received mushroom powder from irradiated mushrooms, while the control group received powder from mushrooms that had not been exposed to UV-light. The daily amount of vitamin D_2_ in the diet of the first group was 1 µg per day. All of the animals were sacrificed at the end of the study to determine femur bone mass density and the serum 25(OH)D levels and compare the results to a control group. Significant effects on both femur bone mass density and serum 25(OH)D concentrations were observed for the study group receiving UV-irradiated mushroom powder. For the serum level, the difference was almost 20-fold, with a vitamin D_2_ concentration of 129 nmol/L in the verum group, while it was only 6.06 nmol/L in the control group after 4 weeks [88]. A similar experiment was also published a few years later for button mushrooms which had been enriched in vitamin D_2_ by UV-light. The mushrooms were grounded and freeze-dried after UV treatment before resuspension in saline. Animals received a daily single peroral application of 50, 100 or 200 mg/kg bodyweight of the resuspended powder and were sacrificed after 3 weeks of application. Plasma 25(OH)D_2_ concentrations were determined for each study group and showed a significant, dose-dependent increase compared with the control group. Even the lowest dose of 0.88 µg of vitamin D_2_ per 50 mg/kg bodyweight was sufficient for the determined effect [66].

Few studies are available assessing baker’s yeast (*S. cerevisiae*) as another potential source of vitamin D_2_ for nutritional supplementation. In 2011, a study investigating the bioavailability of vitamin D_2_ from D_2_-rich yeast baked into bread was published [89]. Vitamin D-deficient rats were fed a diet containing 0.625, 2.5, 5 or 25 µg vitamin D_3_ or vitamin D_2_ from the enriched bread for 7 weeks. The 25(OH)D plasma levels as well as femur bone density and mechanical properties of the femur were utilized to assess the vitamin D status of the animals. The results showed that the plasma 25(OH)D levels did increase for the bread-fed animals. However, the effect was not as pronounced as for the animals receiving the crystalline vitamin D_3_, reaching only approximately 50% of the 25(OH)D plasma level of the D_3_ group when applying the fortified bread. An interesting finding is a curvilinear relationship between the dietary vitamin D supplementation and plasma 25(OH)D concentration. This was determined to be between 2.5 and 5 µg/kg diet for vitamin D_2_ [89], while the inflection point is at around 5 µg/kg diet for vitamin D_3_ [90].

A study in humans on the effect of vitamin D-fortified yeast to produce bread or other baking goods was published in 2016. In this study, 33 Finnish women aged 20 to 37 received either a vitamin D_2_ or vitamin D_3_ supplementation or D_2_-fortified bread. Each participant received approximately 25 µg vitamin D_2_ or vitamin D_3_ with their daily supplementation. Serum samples were taken at initiation of the study, as well as after 4 and 8 weeks of supplementation. The strongest effect on serum 25(OH)D was observed for the vitamin D_3_ supplementation, followed by a slightly lesser extent by the vitamin D_2_ supplementation. The effect of the vitamin D_2_-fortified bread on serum 25(OH)D was only modest compared with the placebo group, which did not receive any supplementation [91].

A study on the safety, bioavailability, and efficacy of vitamin D_2_ from light-exposed edible mushrooms in rats was published by Calvo et al. [92]. While this study showed positive effects on the femur density and microarchitecture, the bioavailability was assessed by comparing the blood plasma levels in rats with those in humans without taking into consideration the different required levels of vitamin D in rats and humans, as there is a much higher sensitivity of rats for vitamin D. This is quite evident when the use of vitamin D derivates for pest control in rodents, and especially rats, causing abnormally high calcium and phosphate serum levels and multisystemic mineralization before death is taken into consideration [93]. Even more striking, for some measurements, the effects were more pronounced for the rats fed 5% mushrooms not exposed to UV-light in their diet compared with rats with 2.5% and 5% UV-light-exposed mushrooms in their diet. Therefore, the conclusion of the authors seems misleading: They state that despite the 30-fold excess of vitamin D_2_ over the recommended daily intake and the higher sensitivity of rats towards vitamin D, the safety of vitamin D_2_ could be confirmed. Considering the discussion of the bioavailability of vitamin D_2_ from mushrooms and yeast, this rather points towards a low bioavailability. Therefore, it can be concluded that most of the vitamin D_2_ from the diet is excreted in the faeces together with the undigested membrane and fibre. The lack of a gall bladder (e.g., bile salts) in rats is presumably contributing to this low bioavailability. Similar results were also obtained by Hohman et al. [89]. The effect of vitamin D_2_ substitution by utilising UV-B-treated yeast for bread leavening on the plasma 25(OH)D status was lower compared with the participants receiving similar doses of crystalline vitamin D_3_. One reason for this finding could be a difference in the kinetics of vitamin D_2_ and vitamin D_3_, pointing to the fact that vitamin D_2_ has both a higher turnover rate and a lower capacity to increase and sustain plasma 25(OH)D levels compared with vitamin D_3_. However, these effects can be overcome by a sufficient daily intake of vitamin D, irrespective of the source and type of vitamin D. Another point could be the different vitamin D status of the animals compared with humans. While the animals were significantly vitamin D-deficient at the start of the study, the human participants were at a sufficient vitamin D status, with no participant showing severe vitamin D deficiency. Therefore, it was speculated by Itkonen et al. that the effect of vitamin D_2_ substitution by mushrooms or yeast treated with UV-light is only significant in severely deficient organisms [91]. The higher sensitivity of rats to vitamin D might also contribute to the positive impact of the peroral supplementation.

Lipkie et al. performed a simulated in vitro digestion test with varying breads using vitamin D_2_-fortified yeast to determine the potential uptake of vitamin D_2_ from this matrix [94]. The authors showed that vitamin D_2_ is mostly inaccessible from yeast, as the yeast cells are not lysed or broken up by osmotic, chemical or mechanical stress, and the vitamin D_2_ bound in the membranes remains inaccessible for uptake. Additional processing of the yeast was speculated to enhance digestive release by overcoming the entrapment within the yeast matrix. This hypothesis was tested by Itkonen et al. in a rat model [95]. Rats with an adequate vitamin D status received either 7.5 or 15 µg vitamin D_3_ as supplementation, or 7.5 or 15 µg vitamin D_2_, either as supplementation, in bread with intact yeast cells which had been treated with UV-light to increase vitamin D_2_ or the cell membrane fraction of UV-light-treated yeast cells. No significant difference on serum 25(OH)D concentration was observed between the intact yeast cell and yeast cell membrane fractions. In this study, it becomes once more obvious that substitution using vitamin D_3_ is more effective compared with vitamin D_2_ substitution, and that there may be some intrinsic characteristic of the yeast that reduces the bioavailability of vitamin D_2_, even after disintegration and fractioning of the yeast cells walls [91,95].

The bioavailability of vitamin D_2_ remains to be a controversial topic. The first to propose a lower bioavailability of vitamin D from natural sources compared with supplementation were van den Berg et al. in 1997 [96]. Since then, several large studies, discussed in previous sections, argued for and against this finding. For instance, there are the findings of both Outila et al. and Urbain et al. that showed no difference in bioavailability after ingestion [67,84]. Therefore, in contrast to the findings in yeast, mushrooms seem to provide a higher bioavailability of vitamin D_2_. However, it also has to be kept in mind that in the study of Outila et al. vitamin D_2_ from mushrooms was provided as lyophilised powder [84] pointing towards a strong disintegration of the lipid membranes and fibres. In Urbain et al., vitamin D_2_-fortified mushrooms were prepared as soup, providing very high amounts of vitamin D [67]. It is not clear from the publication if there was any mechanical mixing or blending of the soup during preparation, which could also reduce cell integrity and thereby increase the bioavailability of vitamin D_2_. In Mehrotra et al., soup prepared from fortified mushrooms was not able to positively impact the serum 25(OH)D status [85]. However, studies claiming a high bioavailability of vitamin D_2_ from nutritional sources are more in line with the extensive review by Borel et al., where the effect of other factors such as fiber or fat content, as well as vitamin D-status of the patient on the bioavailability of both vitamin D_2_ and D_3_ were discussed [31]. From this publication, it is apparent that the bioavailability is only marginally affected by the food composition, especially the fat content of the diet. An interesting finding is that 24-hydroxyvitamin D is better absorbed from the gut than vitamin D, and that the age of the person ingesting the food does not seem to have an impact on vitamin D uptake. All in all, a clear distinction between yeast and mushrooms for vitamin D fortification is required.

## 5. Safety Assessment of UV-Irradiated *Saccharomyces cerevisiae*

### 5.1. The European Food Safety Authority Assessment of 2014

In 2014, the European Food Safety Authority (EFSA) published a scientific opinion on the safety of vitamin D-enriched UV-treated baker s yeast *S. cerevisiae* in order to assess “UV-treated baker s yeast” as a novel food ingredient (NFI) [97]. The assessment was required in the context of Regulation (EC) No. 258/97, last amended 18 July 2009, as the British company Lallemand SAS applied for the registration of UV-light-treated baker’s yeast as NFI for the production of bread and baked goods, according to Article 4 of the said regulation. UV-treated yeast does fall under this regulation due to Article 1, Section 2 of Regulation (EC) No. 258/97 which states:

“This Regulation shall apply to the placing on the market within the Community of foods and food ingredients which have not hitherto been used for human consumption to a significant degree within the Community and which fall under the following categories:

[…]

(f) Foods and food ingredients to which has been applied a production process not currently used, where that process gives rise to significant changes in the composition or structure of the foods or food ingredients which affect their nutritional value, metabolism or level of undesirable substances” [97].

In the scientific assessment by the EFSA, it was stated that the applicant was only able to determine vitamin D_2_ and tachysterol by HPLC analysis. The determined amounts were 672 and 825 µg/g of vitamin D_2_ and 140 and 145 µg/g of tachysterol in two commercial batches of UV-treated baker’s yeast. The intended use of the baker’s yeast was to produce a bread product containing 5 µg or 200 IU/100 g, requiring 6.67 mg of the vitamin D_2_ yeast concentrate which contains 30,000 IU/g. An amount of 0.93 µg (corresponding to 37.2 IU) tachysterol/100 g bread would be applied using this NFI. Therefore, the panel concluded that tachysterol was not to be included in the product specification. Data were also provided on the stability of vitamin D_2_ during storage of the NFI, as well as the production and storage of the finished dietary goods. No significant impact on the vitamin D_2_ content in any of the provided products was observed, and therefore no safety concerns regarding degradation products exist. The same conclusion was also drawn for the potential formation of irradiation products of lipids and proteins [98].

The anticipated intake of the NFI and its effect on the overall daily vitamin D uptake was also assessed from the consumption of yeast-leavened bread, rolls, and fine pastry. The Panel utilized the chronic food consumption statistics provided by the EFSA Comprehensive Food Consumption Database [99]. As there was no differentiation in the fine pastry category between goods produced using yeast and those that use other production methods, there was an overestimation of the intake. This was taken as an additional conservative safety measure. The highest potential intakes (97.5th percentile) were 27.2 µg/d for children of age 4 to 10 from Latvia, 36.5 µg/d in adolescents from Germany, and 35.5 µg/d in adults from Latvia. Given the tolerable upper intake levels of vitamin D established by the EFSA of 50 µg/d for children aged 4 to 10 and 100 µg/d for adults, the NFI by itself is not able to reach amounts of concern even in the 97.5th percentile of consumption.

Next to the intake of the NFI through fortified baked goods, the impact on the overall vitamin D intake was assessed to determine if the tolerable upper intake levels of vitamin D could be exceeded. Intake levels from the EFSA NDA Panel of 2012 were utilized [100]. The highest intakes of vitamin D in the 95th percentile were 11.9 µg/d for children (Greece, 90th percentile, 1 to 5 years), 7.7 µg/d for adolescents (Italy, boys, 10 to <18 years), and 16.0 µg/d for adults (Finland, men, 25 to 74 years). When also considering supplements, the intake was determined to be as high as 24.2 µg/d (Ireland, ≥65 years). Therefore, even the additional intake of the NFI would not lead to an excess of the tolerable upper intake levels. After additional assessments of allergenicity, microbiological, and nutritional information and considering the long history of the safe use of yeast and yeast-leavened products, the Panel considered UV-treated baker’s yeast exhibiting an enhanced content of vitamin D_2_ as safe under the intended conditions of use [98].

### 5.2. Update of the Safety Assessment of UV-Irradiated Baker’s Yeast

Since the safety assessment by the EFSA in 2014, there were some additional findings regarding the composition of yeast and mushrooms after UV irradiation. Furthermore, there were aspects missing in the assessment that, in the opinion of the author, should have been considered.

First, it needs to be checked if any update in the chronic food consumption patterns have been reported [99]. When assessing the data provided, it becomes clear that since 2014 four additional surveys have been performed, two coming from Austria and one each from Portugal and Slovenia. In none of these additional surveys, a higher intake than the previously reported highest intakes of 544 g of bread and baked goods per day for children aged 4 to 10 years in Latvia, and 710 g of bread and baked goods per day in adults, also in Latvia, were determined. Therefore, these values are not changed for the upcoming assessment of the potential daily intake compared with the EFSA assessment of 2014 [98]. However, the consideration remains questionable in view of new data.

Philips et al. already showed in 2012 that the analytics of vitamin D is not trivial, and that special care has to be taken when determining the content of all the different vitamin D variants in mushrooms [42]. This became even more evident in the publication of Wittig et al. in 2013 when determining the amounts of vitamin D_2_ and vitamin D_4_ next to their respective previtamins, lumisterols, and tachysterols [44]. Wittig et al. were able to show that even under near-optimal reaction conditions for vitamin D formation, only between 37% to 55% of the yield are the respective vitamin D variants, while the remaining fraction is distributed among the respective side products. This discrepancy is based on the different extraction methods utilized, with hot alkaline hydrolysis leading to the highest vitamin D_2_ yield compared with the cold alkaline hydrolysis or ultrasound assisted extraction [44]. Therefore, it is surprising that neither the formation of vitamin D_4_ and any of its side products nor the formation of lumisterol was observed in the analytical data provided to the EFSA for the safety assessment of UV-treated baker’s yeast. This becomes even more striking when considering the chosen production conditions of the applicant. As disclosed in the safety assessment, the yeast crème produced by fermentation is irradiated while being pumped through tubes, using UV-lights with a maximum emission wavelength at 250 nm. The determined ratios of vitamin D to tachysterol are between 4.8:1 and 5.7:1. As previously discussed, the optimal wavelength for vitamin D generation is at 295 nm, while shorter wavelengths favour the generation of tachysterol. The ratio of vitamin D to tachysterol as observed in Wittig et al. is 5:1 under optimal vitamin D-producing conditions. Therefore, the production ratio for the UV-light-treated baker´s yeast is in a similar range, although one could have expected a lower ratio, reflecting a favoured tachysterol formation under the reaction conditions chosen by the applicant.

Under these circumstances, it seems reasonable to re-investigate the provided amounts of vitamin D_2_, as well as vitamin D_4_ and all their respective photoproducts in yeast-leavened products prepared using the NFI. The hypothetical ratio of all the compounds will be set according to Wittig et al. for all of the three extraction methods, and the expected amount calculated based upon fortification of 5 µg vitamin D_2_ in 100 g baked goods using the NFI. The results are given in Table 3, Table 4 and Table 5.

When taking the sum of all the listed compounds in Table 3, Table 4 and Table 5, the intake from baked goods can reach between 10.48 and 15.72 µg of vitamin D-associated substances in 100 g of bread, corresponding to 419.2 to 629 IU per 100 g of bread. This clearly exceeds the 200 IU which are considered to be acceptable for the fortification of yeast-leavened baked goods. The same acceptable intake level was also determined by an assessment of the US FDA [101], while the recommendation by Health Canada Department of Health, Food, and Drug Regulation is only 90 IU per 100 g yeast-leavened bread [102].

Given the excess of 110% to 215% of the side products compared with vitamin D_2_, the sum of these compounds should be taken into consideration of the safety assessment. However, similar to the different biological activities seen or proposed for the different vitamin D isomers, the biological activity of the photo-isomers is not identical to the one of vitamin D_2_. Therefore, it would be of interest to find a way to set the biological activity of the photo-isomers into a relationship with the biological activity of vitamin D_2_ using equivalence factors comparable with the utilisation equivalence factors for, e.g., dioxin congeners to determine the toxicity of a mixture of different dioxin congeners [103]. Vitamin D_2_ would in this case act as the “lead substance” with its biological activity set to 100%. The biological activity of mixtures of vitamin D isoforms and photoproducts with known composition will be determined by multiplying the concentration of each compound with its equivalence factor and summing up all of the resulting equivalence concentrations. This approach of course requires data on the biological activity and kinetics of all the eight different isomers in relation to vitamin D_2_. While the activities of vitamin D_2_ and vitamin D_4_ with respect to each other are known, the other activities need to be obtained from different sources. Binding affinities of the different compounds to VDR or their capability to modify the transcription of enzymes involved in vitamin D metabolism could act as a mean to determine the biological activity of vitamin D isomers. However, Chen et al. have shown that the binding affinity is not necessarily correlated to the biological activity [104]. Conversion of the different isoforms into other forms induced by either heat or UV light could also cause the biological activity of initially inactive compounds [77]. The biological activity of previtamin D by itself is very low (in the range of 2%), as shown by utilising locked previtamin compounds and testing their biological activity both in vivo and in vitro [105,106]. On the other hand, 90% of previtamin D is converted to vitamin D in human skin within 20 h in a temperature-dependent manner, and an equilibrium constant of K = 11.44 at 37 °C [107]. It could be shown that amphophilic interactions with membrane compounds are able to stabilise previtamin D and prevent its conversion to vitamin D [108]. Therefore, interactions with long-chain fatty acids present in the plasma membrane favour the formation of vitamin D from its precursor. However, even in unphysiological conditions in pure n-hexane, 40% of previtamin D are converted to vitamin D in 20 h [107]. Therefore, this conversion rate will be taken as a proxy for previtamin D activity, in relation to the activity of vitamin D_2_ or vitamin D_4_, respectively.

The impact of oral uptake of lumisterol in mice has been studied and published in detail recently [77]. The results can of course not be transferred to humans directly. However, an interesting finding was that the UV light and temperature-dependent conversion of lumisterol to vitamin D, next to the impact on the vitamin D metabolism, can be observed. There is already a small conversion of lumisterol to vitamin D in the diet, but this is less than 1%. As the conversion of lumisterol in vivo cannot be quantified, and the impact on the vitamin D metabolism is only seen at significantly higher lumisterol uptake levels than present in the food products, the biological activity of lumisterol is set to zero.

Hardly any information on the biological activity of tachysterol are available. It is actually described as chemically inert and with no biological activity [75]. Similar as for lumisterol, the biological activity is set to zero.

Vitamin D_4_ was assessed to have approximately 60% of the biological activity of vitamin D_3_ [42]. Therefore, any activities of the respective photo-isomers have to be adjusted to this reduced biological activity under the assumption that vitamin D_2_ and vitamin D_3_ are equivalent in their biological activity. The activity of previtamin D_4_ would consequently be 24% of the vitamin D_2_ activity, while the activities of lumisterol_4_ and tachysterol_4_ are both set to zero, as for their vitamin D_2_ analogues. The detailed calculations are shown in columns 5 to 7 of Table 3, Table 4 and Table 5.

Utilising these activities, the equivalent international units would be 5.98 to 7.37 µg or 239 to 295 IU in 100 g of yeast-leavened baked goods. This increase over the aspired vitamin D_2_ content in the NFI can be seen as negligible, especially considering the impact on the overall daily vitamin D uptake and the distance between the 95th percentile of intake and the tolerable upper intake levels for both children and adults. The highest potential intake in the 97.5th percentile of vitamin D_2_ from bread and other baked goods was assessed to be 35.5 µg, corresponding to a consumption of 710 g of bread and baked good using the NFI in the Scientific Opinion by the EFSA [98]. Under the assumption of the highest amount of vitamin D and its photoproducts from the previous assessment, the actual intake for the 97.5th percentile would not be 35.5 µg, but rather 52.3 µg for an adult, and 40.1 µg for children in the 97.5th percentile of bread and baked goods consumption of 544 g/d. Added to the highest intakes of vitamin D in the 95th percentile of 11.9 µg/d for boys aged 1 to 3 years, 16.0 µg/d for adults without supplementation, and 24.2 µg/d for adults with supplementation. This results in hypothetical daily intakes of 52.0 µg/d for children and 68.3 or 76.5 µg/d in adults, depending on nutritional supplementation. While the amount in adults is in any case still significantly below the tolerable upper intake level of 100 µg per day, the amount of 52.0 µg per day for children exceeds the tolerable upper intake level of 50 µg per day for this population cohort. The daily intake level from food sources in children can be even higher in the case of substitution of vitamin D, for which no information is available, in contrast to the adult vitamin D substitution. Therefore, in the case for children, the results from nutritional intake should be multiplied by 1.5, according to the data provided in the EFSA Scientific Opinion.

The results for the daily intake could even be too low, as hypothetically present amounts of both lumisterol and tachysterol in the final product were not included in the assessment. This exclusion could be challenged, especially for the assumption that tachysterol and lumisterol show no biological activity. While tachysterol itself is chemically inert and does not show any biological activity, it can isomerize back to its corresponding previtamin under UV-light at a wavelength of 315 to 340 nm. Tachysterol can thereby act as both a side product during high levels of irradiation and inert storage molecule for previtamin formation at low previtamin D levels or unfavourable irradiation conditions. On the other hand, the quantum yield of this reaction is low. From both spectroscopic as well as computational simulation data, the occurrence of this backward conversion upon photoexcitation is however assessed to be small, and does not contribute significantly to previtamin D formation [75]. On the other hand, tachysterol has been shown to be a substrate for the D-25-hydroxylase in isolated keratinocytes, where 25(OH)tachysterol could be recognized by VDR, thereby exerting biological activity. However, this activity was seen to be small. Despite the high binding affinity of tachysterol for VDR, the biological activity was still low [104]. Our own data from osteocyte cell culture experiments were able to show the biological activity of tachysterol and 25(OH)tachysterol, respectively (unpublished data). Nevertheless, tachysterol was excluded from the assessment. A similar case can be made for lumisterol, which was shown to strongly influence the metabolism of vitamin D in vivo when applied at high amounts, while also to have the ability to be converted to vitamin D [77].

Next to lumisterols and tachysterols, the production of other photoproducts is potentially also possible in irradiated yeast and mushrooms. As shown during the discovery and characterization of these compounds in the 1970s, their occurrence is low, and significant amounts are only achieved at high concentrations of previtamin D in organic solvents [10,79]. Therefore, these photoproducts can be excluded from the safety assessment, as their abundance in irradiated food is small, determined to be at approximately 0.1% even after strong overirradiation. This exclusion from toxicological considerations was already proposed shortly after their discovery [109]. Next to their low abundance, their chemical structure also makes these compounds unlikely to cause significant toxicity. Although the exact mechanism of vitamin D toxicity is not clear yet, an involvement of binding to either DBP, VDR or both seems very likely. For efficient binding, the orientation of the hydroxyl groups of vitamin D or its hydroxylated products is critical [21]. In particular, the A-ring region with its 3′-hydroxyl group is of importance here. As neither of the suprasterols or toxisterols show a high structural similarity to the A-ring region of vitamin D due to the formation of alternative structures after irradiation of the triene region, the binding of these compounds to either DPB or VDR is not considered to be relevant, and none of these compounds were therefore included in the assessment.

The consideration for the suprasterols and toxisterols also applies to pyrocalcitol and isopyrocalcitol, as the formation of these products only occurs at temperatures above 100 °C, and therefore is unlikely to occur under normal cultivation, irradiation, and transport conditions of the NFI. However, there can be a significant formation of pyrocalcitol and isopyrocalcitol during the processing of food products utilising the NFI, given the high amount of previtamin D. However, adverse effects for the consumer seem unlikely given the molecular structure of these side products, similar to the assessment of photoproducts in the previous section. Additional thought should be given to the effect in processed food containing vitamin D-fortified raw materials, as for yeast in bread. The stability of vitamin D in its source materials is considered to be high, but can be influenced by both temperature and UV irradiation, shifting the equilibrium towards precursors and side products. Additionally, it was shown that the equilibrium can also be shifted in the presence of iodine, oxygen, and at low pH. While iodine causes *cis/trans* isomerisation, resulting in *trans*-vitamin D_3_ and tachysterol, acidic conditions give rise to the formation of iso-tachysterol [110].

One can of course question the inclusion of vitamin D_4_ in the assessment. Previous experiences during the safety evaluation of ionising radiation for control microbial growth in food have shown that animal toxicity studies were too insensitive to detect the effects of putative radiolytic products that may be produced at low concentrations in the food. This position is further reflected in the FDA’s opinion on the generic use of UV-C-light for germicidal control of microbial growth in food. Therefore, hazard identification and characterization was limited to an evaluation of the compositional changes in mushrooms, occurring due to the intended use of UV-light for the production of vitamin D in mushrooms and in consideration of the nutritional and toxicological impact of introducing additional dietary sources of vitamins D_2_ and D_4_ to the food supply. Due to a lower bioavailability, lower activity, and faster degeneration, the presence of vitamin D_4_ was considered nutritionally and toxicologically insignificant [82]. However, a complete exclusion was not considered for the present safety assessment, as there has been a significant finding of vitamin D_4_ in fungi after UV irradiation, and the proven effect of vitamin D_4_ upon uptake in animal models.

For this assessment, the biological activity of vitamin D_2_ was considered to be equivalent to the activity of vitamin D_3_, also based on an unpublished human study utilising UV-B-irradiated wheat germ oil (submitted paper). However, there are findings that vitamin D_2_ could only have 30% to 50% of the biological activity of vitamin D_3_, which has even led to recommendations to abstain from using vitamin D_2_ for vitamin supplementation despite the recognized fact of the strong impact of vitamin D_2_-fortified products in rickets prophylaxis in the USA [111]. In combination with the determined low bioavailability of vitamin D from yeast-leavened products both in vivo and in an artificial digestion model, one can wonder if an enrichment of yeast with vitamin D_2_ is worthwhile for the economic burden for the customer [89,94]. Even in the case of lifestyle choices regarding nutrition, the alternative of wild or UV-light-treated mushrooms from cultivation seem to be a more efficient choice, at least with regards to bioavailability of vitamin D_2_.

The finding that there is a potential daily intake of vitamin D in a population cohort that exceeds the tolerable upper intake level gives rise to concern. There is a long list of conservative assumptions regarding the assessment, which include the fact that all of the consumed bread and fine pastry goods would be prepared with UV-irradiated yeast and that the amount of vitamin D isomers and their respective photoproducts correspond to the ones seen by Wittig et al. in their analytics, including a bioavailability that corresponds to the harsh reaction conditions utilized for sample preparation. The biological activity of vitamin D_2_ itself is also questioned in recent years, which is also not reflected in the present assessment. Furthermore, degradation processes of vitamin D_2_ during baking, cooking, and storage of the finished food product were also not included. Lavelli et al. showed that temperature, pH of the dough, and water activity can have a high impact on the isomerisation of vitamin D_2_ to isotachysterol, providing an additional option for a reduction of the physiological activity of vitamin D_2_ [112]. Therefore, it seems unlikely that the finding of this assessment with all its assumptions would reflect a health risk for the population cohort in question. Nevertheless, it shows that there is currently not enough available information regarding the composition of UV-irradiated baker’s yeast. Therefore, a new assessment utilising the available analytical and physiological knowledge seems required.

## 6. Conclusions

The consumption of foods prepared with UV-irradiated baker’s yeast to increase the vitamin D content was considered to be safe when examining the production of vitamin D and its associated photoproducts by the EFSA in 2014. This review, utilising a hypothetical assessment of the vitamin D_2_ and vitamin D_4_ content as well as their associated photoproducts after UV irradiation, concluded that there is a potential risk for a population cohort with a high intake of yeast-leavened baked goods. While there are several assumptions that can be challenged considering recent findings on vitamin D bioavailability and biological activity, a final risk cannot be excluded. The scientific opinion of the EFSA should have put a higher emphasis on the generation of the photoproducts and their occurrence in yeast, utilising the available data both from UV irradiation of yeast and mushrooms, especially when the significant challenges regarding vitamin D analytics from biological matrices were already known at the time. In the future, the proposed approach to determine the occurrence of all the photoproducts in the irradiated product and to include the biological activity of these photoproducts should be considered. As the focus of this review was purely on vitamin D and its photoproducts, no conclusions can be drawn for the other aspects addressed in the safety assessment, e.g., microbial, allergic or nutritional aspects of the NFI.

## 7. Literature Search Strategy

The initial literature search for this review was conducted on 16 August 2021 using PubMed. Seven different searches were performed, and the search terms as well as the number of results are shown in Table 6.

All of the search results were reviewed individually, and the relevance of each hit was assessed by checking the relevance for the review based on the abstract. All of the findings deemed relevant were included in a literature database, thereby identifying duplicates. Additional literature was identified by cross-referencing of the previously identified literature sources.

## Figures and Tables

**Table 1:** Diagnostic cut-off points for 25-hydroxy-vitamin D (25(OH)D) concentrations, providing both commonly utilised 25(OH)D plasma concentrations. Table adapted from [34,35].

Category	nmol/L	µg/L
Deficiency	<30	<12
Insufficiency	30–50	12–20
Sufficient	>50	>20
Excess	>250	>100
Intoxication	>375	>150

**Table 2:** Overview of vitamin D_2_ yields after UV irradiation, utilising different mushrooms, provided forms, wavelengths, and light intensities. Vitamin D_2_ contents marked with a star refer to the fresh, undried state.

Mushroom	Latin Name	Form	UV Wavelength	Irradiation Dose, J/cm^2^	Yield, µg/g Dryweight	Time of Exposure, min	Source
Shiitake	*Lentinus edodes*	powder in ethanol	UV-C	24	629	120	[59]
Oyster	*Pleurotus ostreatus*	powder in ethanol	UV-C	24	275	120	[59]
Funnel chanterelle	*Cantharellus tubaeformis*	lyophilized	254 nm	379	14	120	[60]
White Button Mushroom	*Agaricus bisporus/white*	fresh	254 nm	379	10	120	[60]
White Button Mushroom	*Agaricus bisporus/white*	whole pileus	UV-B	2	13.03	not provided	[61]
White Button Mushroom	*Agaricus bisporus/white*	whole gill	UV-B	2	16.7	not provided	[61]
Shiitake	*Lentinus edodes*	pileus layer	UV-B	2.5	37	not provided	[61]
Shiitake	*Lentinus edodes*	middle layer	UV-B	2.5	69	not provided	[61]
Shiitake	*Lentinus edodes*	gill layer	UV-B	2.5	106	not provided	[61]
White Button Mushroom	*Agaricus bisporus/white*	fresh, growing	UV-B	0.25	1.64	4320	[62]
White Button Mushroom	*Agaricus bisporus/white*	fresh	305 nm	1.5	7.43	25	[63]
White Button Mushroom	*Agaricus bisporus/white*	fresh, growing	UV-B	3.24	0.73	3 × 60	[64]
Brown Button Mushroom	*Agaricus bisporus/brown*	fresh, growing	UV-B	3.24	0.88	3 × 45	[64]
Oyster	*Pleurotus ostreatus*	fresh, growing	UV-B	3.24	2.28	3 × 90	[64]
White Button Mushroom	*Agaricus bisporus/white*	fresh	UV-B	1.08	4.1	not provided	[65]
White Button Mushroom	*Agaricus bisporus/white*	fresh	UV-C	0.25	23	60	[66]
White Button Mushroom	*Agaricus bisporus/white*	sliced, dried	UV-B	0.53	67.1	not provided	[67]
Shiitake	*Lentinus edodes*	fresh	UV-B	3.53	6.05	120	[68]
Oyster	*Pleurotus ostreatus*	fresh	UV-B	3.53	4.4	120	[68]
White Button Mushroom	*Agaricus bisporus/white*	fresh	UV-B	3.53	7.8	120	[68]
Enoki	*Flamulina velutipes*	fresh	UV-B	3.53	0.68	120	[68]
Woddy mushroom	*Auricularia auricula*	fresh	UV-B	3.53	0.68	120	[68]
White Ear mushroom	*Tremella fuciformis*	fresh	UV-B	3.53	0.32	120	[69]
Portabella mushroom	*Agaricus bisporus/white*	fresh	UV-B	3.53	6.76	120	[68]
brown beech mushrom	*Hypsizugus tessulatus*	fresh	UV-B	3.53	3.07	120	[68]
Abalone mushroom	*Pleurotus ostreatus*	fresh	UV-B	3.53	4.35	120	[68]
White Button Mushroom	*Agaricus bisporus/white*	cap	UV-C	0.2	1340	3	[57]
White Button Mushroom	*Agaricus bisporus/white*	stem	UV-C	0.2	1170	3	[57]
Brown Button Mushroom	*Agaricus bisporus/brown*	cap	UV-C	0.2	950	3	[57]
Brown Button Mushroom	*Agaricus bisporus/brown*	stem	UV-C	0.2	1050	3	[57]
White Button Mushroom	*Agaricus bisporus/white*	fresh	UV-B	1.47	12.48	120	[51]
	*Agaricus bitorquis*	fresh	UV-C	1.47	5.32	120	[51]
Shiitake	*Lentinus edodes*	fresh	UV-B	1.47	6.58	120	[51]
Straw mushrooms	*Volvariella volvacea*	fresh	UV-B	1.47	7.58	120	[51]
Oyster	*Pleurotus ostreatus*	fresh, from bottom	UV-B	4.14	160	60	[58]

**Table 3:** Yield from hot alkaline hydrolysis of oyster mushrooms after UV irradiation, determined by HPLC. The yields for the different identified vitamin D isomers as well as precursors and photoinduced side products are provided, as well as the percentage of each compound normalised to the identified vitamin D_2_ amount. The hypothetical amounts of all the precursors and photoinduced side products in vitamin D_2_-fortified bread based on an amount of 5 µg per 100 g bread is provided.

Compound	Hot Alkaline Hydrolysis, µg/g, µg/g [44]	Percentage, Vitamin D_2_ Set to 100	Hypothetical Amount, µg/100 g Bread	Biological Activity, %	Equivalence Amount, µg/100 g Bread	Percentage, Normalised to Vitamin D_2_, %
previtamin D_2_	32.07	22.69	1.13	40	0.45	9.08
tachysterol_2_	30.36	21.48	1.07	0	0.00	0.00
**vitamin D_2_**	**141.32**	**100.00**	**5.00**	**100**	**5.00**	**100.00**
lumisterol_2_	50.67	35.85	1.79	0	0.00	0.00
previtamin D_4_	5.23	3.70	0.19	24	0.04	0.89
tachysterol_4_	5.11	3.62	0.18	0	0.00	0.00
vitamin D_4_	22.72	16.08	0.80	60	0.48	9.65
lumisterol_4_	8.59	6.08	0.30	0	0.00	0.00
Sum	296.07		10.48		5.98	119.61

**Table 4:** Yield from cold alkaline hydrolysis of oyster mushrooms after UV irradiation, determined by HPLC. The yields for the different identified vitamin D isomers as well as precursors and photoinduced side products are provided, as well as the percentage of each compound normalised to the identified vitamin D_2_ amount. The hypothetical amounts of all the precursors and photoinduced side products in vitamin D_2_-fortified bread based on an amount of 5 µg per 100 g bread is provided.

Compound	Cold Alkaline Hydrolysis, µg/g [44]	Percentage, Vitamin D_2_ Set to 100	Hypothetical Amount, µg/100 g Bread	Biological Activity, %	Equivalence Amount, µg/100 G Bread	Percentage, Normalised to Vitamin D_2_, %
previtamin D_2_	68.40	86.81	4.34	40	1.74	34.73
tachysterol_2_	24.07	30.55	1.53	0	0.00	0.00
**vitamin D_2_**	**78.79**	**100.00**	**5.00**	**100**	**5.00**	**100.00**
lumisterol_2_	41.12	52.19	2.61	0	0.00	0.00
						
previtamin D_4_	11.35	14.41	0.72	24	0.17	3.46
tachysterol_4_	4.30	5.46	0.27	0	0.00	0.00
vitamin D_4_	12.08	15.33	0.77	60	0.46	9.20
lumisterol_4_	7.58	9.62	0.48	0	0.00	0.00
						
Sum	247.69		15.72		7.37	147.38

**Table 5:** Yield from ultrasound assisted extraction of oyster mushrooms after UV irradiation, determined by HPLC. The yields for the different identified vitamin D isomers as well as precursors and photoinduced side products are provided, as well as the percentage of each compound normalised to the identified vitamin D_2_ amount. The hypothetical amounts of all the precursors and photoinduced side products in vitamin D_2_-fortified bread based on an amount of 5 µg per 100 g bread is provided.

Compound	Ultrasound Assisted Extraction, µg/g [44]	Percentage, Vitamin D_2_ Set to 100	Hypothetical Amount, µg/100 g Bread	Biological Activity, %	Equivalence Amount, µg/100 g Bread	Percentage, Normalised to Vitamin D_2_, %
previtamin D_2_	59.02	84.06	4.20	40	1.68	33.62
tachysterol_2_	16.65	23.71	1.19	0	0.00	0.00
**vitamin D_2_**	**70.21**	**100.00**	**5.00**	**100**	**5.00**	**100.00**
lumisterol_2_	37.39	53.25	2.66	0	0.00	0.00
previtamin D_4_	7.73	11.01	0.55	24	0.13	2.64
tachysterol_4_	2.72	3.87	0.19	0	0.00	0.00
vitamin D_4_	9.45	13.46	0.67	60	0.40	8.08
lumisterol_4_	5.58	7.95	0.40	0	0.00	0.00
Sum	208.75		14.87		7.22	144.34

**Table 6:** Used search terms for the initial literature search, also showing the number of hits for each search term, and the number of used literature sources finally utilised.

Search Term	Hits	Used
mushroom vitamin D UV	51	19
cerevisiae vitamin D UV-light	10	2
cerevisiae vitamin D enrichment	1	0
tachysterol	38	7
tachysterol toxic	2	0
mushroom tachysterol	1	1
tachysterol food	8	2
